# Supplementary material for: Rational Design of Unsaturated, Thioether Ionizable Lipids for Enhanced In Vivo mRNA Delivery
Source: Adv Healthc Mater. 2025 May 5;14(15):2501037. doi: 10.1002/adhm.202501037 (PMC12147982; doi:10.1002/adhm.202501037)
Supplement: Supplementary file 1 — Supporting Information [file ADHM-14-0-s001.docx]

Supporting information

Rational Design of Unsaturated, Thioether Ionizable Lipids for Enhanced *In vivo* mRNA Delivery

Authors

Eleni Samaridou^1^, Johanna Simon^1^, Moritz Beck-Broichsitter^1^, Gary Davidson^2,3^*, Pavel A. Levkin^2,3^*

Affiliations:

^1^Merck KGaA, Frankfurter Str. 250, 64293 Darmstadt, Germany

^2^ScreenFect GmbH, Lauterstr. 5a, 76344 Eggenstein-Leopoldshafen, Germany

^3^Institute of Biological and Chemical Systems (IBCS-FMS), Karlsruhe Institute of Technology (KIT), Kaiserstrasse 12, 76131 Karlsruhe

*Emails: [gary.davidson@kit.edu](mailto:gary.davidson@screenfect.com), pavel.levkin@screenfect.com,

Content

[Synthesis and characterization of thioether lipids. 2](#_Toc181253528)

[Biological testing of LNPs containing novel thioether lipids. 10](#_Toc181253529)

## Synthesis and characterization of thioether lipids.

Reagents used for the synthesis:

Hept-1-yne, potassium thioacetate (KSAc), ammonia (7M), mesyl chloride, N,N-dimethylethane-1,2-diamine, N,N-dimethylaminoethylamine, 1-octadecanethiol, BOC-protected amines (various), pentylchloride, 5% Pd/CaCO₃, Pd/BaSO₄, ethanol, methanol,

petroleum ether, dry methanol were purchased from Fisher Scientific (Schwerte, Germany).

Dodecyl iodide was purchased from Sigma (Schnelldorf, Germany). N-methyl-1,2-ethylenediamine and trifluoroacetic acid (TFA), dichloromethane (DCM), anhydrous dimethylformamide, diethyl ether, acetic acid, sodium sulfate (Na₂SO₄), celite (for filtration), molecular sieves (for drying solvents) were bought from Carl Roth (Karlsruhe, Germany). Lindlar catalyst (5% Pd/CaCO₃ poisoned with Pd or Pd/BaSO₄) and sodium methylate were obtained from TCI (Eschborn, Germany). Quinoline was received from Merck KGaA (Darmstadt, Germany). Hydrogen gas was purchased from Air Liquide (Ludwigshafen, Germany). 2-(4-Iodobutoxy)tetrahydro-2H-pyran (1) and dibromide (25) were obtained from GenoSynth GmbH (Berlin, Germany). Oleyl alcohol, linoleyl alcohol ((9Z,12Z)-octadecadien-1-ol), N^1^-BOC, N^2^-methylethane-1,2-diamine, N-methyl-N-pentylamine, BOC-protected compound 30, Hexynol 33, dodecyl iodide, n-butyllithium, triisopropylsilylchloride were purchased from Sigma (Germany).

Methods

1. Lipid synthesis

*Synthesis of A1C11_D5*

Scheme 1. Synthesis scheme of A1C11_D5

The synthesis began by alkylation of the hept-1-yne (2) with 2-(4-iodobutoxy)tetrahydro-2H-pyran (1) according to a published procedure.^1^ This resulted in the formation of the protected alkyne 3. Subsequent acid-catalyzed deprotection of the tetrahydropyranyl (THP) group afforded the corresponding alcohol 4.

Alkyne 4 was then subjected to Lindlar reduction to selectively obtain the alkene 5. The crude alkyne 4 (13.98 g, 83.08 mmol, 1.0 eq.) was added to a 1L three-necked reaction flask, followed by the addition of ethanol (500 mL) as solvent. To the reaction mixture, 5% Pd/CaCO_3_ (700 mg, poisoned with Pd), Pd/BaSO4 (10% Pd, 700 mg), and quinoline (2 mL, 17.45 mmol, 0.21 eq.) were introduced under a nitrogen atmosphere. The system was purged with hydrogen gas, and the reaction was stirred at room temperature until completion. Upon completion, the reaction mixture was filtered through celite and concentrated under reduced pressure. The crude product was purified by flash chromatography, yielding the alkene 5 as a yellow oil with a 48% yield.

Alcohol 5 was mesylated using mesyl chloride to produce the mesylated compound 6. Subsequently, 6 was reacted with potassium thioacetate (KSAc) to yield AcS-derivative 7. The detailed procedure is as follows: In a 100 mL round-bottomed flask equipped with magnetic stirring, mesylate 6 (11.99 g, 48.3 mmol, 1 eq) was dissolved in anhydrous DMF (50 mL). Potassium thioacetate (11 g, 96 mmol, 2 eq) was added to the reaction mixture in one portion, and the reaction was stirred overnight at room temperature. Upon completion, the mixture was poured into water (500 mL) and extracted with diethyl ether (2 x 200 mL). The organic layers were dried over sodium sulfate (Na₂SO₄), and the solvent was removed under reduced pressure to afford the crude product 7.

The obtained thioester 7 was hydrolyzed to yield thiol 8 according to the procedure: In a 100 mL round-bottomed flask with magnetic stirring, alkenyl thioacetate (11.2 g, 49 mmol, 1 eq) was dissolved in methanol (MeOH) containing 7 M ammonia (28 mL). The reaction was stirred at room temperature overnight. After completion, the reaction mixture was concentrated under reduced pressure and purified by flash chromatography, eluting with neat petroleum ether (PE), to yield thiol 8 as a colorless oil (76% yield).

Compound 9 was synthesized by coupling alkenyl thiol 8 with dibromide 25. In a 500 mL round-bottomed flask with magnetic stirring, sodium methylate (2.17 g, 40.14 mmol, 2.2 eq) was suspended in dry methanol (80 mL, dried over molecular sieves). Alkenyl thiol 8 (7.48 g, 40.14 mmol, 2.2 eq), dissolved in diethyl ether (80 mL, dried over molecular sieves), was added dropwise to the sodium methylate suspension. The mixture was stirred for 30 minutes at room temperature. Dibromide 25 (5 g, 18.24 mmol, 1.0 eq) was then added in one portion, and the reaction was stirred overnight while monitoring progress by TLC. Afterward, acetic acid (1 mL) was added, and the solvent was evaporated under reduced pressure to yield the crude ester 9.

The crude methyl ester 9 was directly subjected to amidation with neat N,N-dimethylaminoethylamine. The ester was dissolved in the amine, and the reaction mixture was heated at 60°C overnight. Upon completion, the solvent was evaporated under reduced pressure, and the crude product was purified by flash chromatography to afford the target lipid A1C11_D5.

*Synthesis of A1C18_D9*

Scheme 2. Synthesis scheme for A1C18_D9

A1C18_D9 was synthesized following a procedure analogous to that used for A1C11_D5, beginning with commercially available oleyl alcohol 10. The alcohol was first mesylated using mesyl chloride, yielding mesylated intermediate 11. This intermediate was then treated with potassium thioacetate (KSAc), resulting in the formation of the AcS-derivative 12. Subsequent hydrolysis of compound 12 produced thiol 13, which was further reacted with dibromide 25. The final step involved direct amidation with dimethylaminoethyl, leading to the target lipid, A1C18_D9.

*Synthesis of A1C18_D9_D12*

Scheme 3. Synthesis scheme of A1C18_D9_D12

A1C18_D9_D12 was synthesized following the same procedure used for A1C11_D5, starting from linoleyl alcohol 15 ((9Z,12Z)-octadecadien-1-ol) as the initial material.

*Synthesis of A1C18*

Scheme 4. Synthesis scheme of A1C18

A1C18 was synthesized following a published two-step procedure. In the first step, alkyne 20 was reacted with 1-octadecanethiol through a UV-induced thiol-yne reaction. This was followed by the amidation of the resulting carboxylic acid with the corresponding amine, as previously described,^2^ to give A1C18*.*

*Synthesis of precursors, dibromide 25, and amines 28 and 32*

Scheme 5. Synthesis scheme of dibromide 25, and amines 28 and 32

Dibromide 25 was synthesized by addition of brom to alkene 24 following a standard procedure.^3^

Amine 28 was prepared by alkylating N^1^-BOC, N^2^-methylethane-1,2-diamine 26 with pentylchloride, followed by BOC deprotection using trifluoroacetic acid (TFA) in dichloromethane (DCM), yielding amine 28.

In a similar manner, amine 32 was synthesized by alkylating *N*-methylpentan-1-amine 29 with the BOC-protected compound 30. Subsequent BOC deprotection yielded the desired amine 32.

*Synthesis of A1C18_D5, A2C18_D5, A3C18_D5 and A4C18_D5*

Scheme 6. Synthesis schemes for A1C18_D5, A2C18_D5, A3C18_D5 and A4C18_D5

Hexynol 33 was initially protected with a triisopropylsilyl (TIPS) group, followed by alkylation with dodecyl iodide (C₁₂H₂₅I) using n-butyllithium to yield alkyne 35. Afterward, the TIPS group was removed to produce octadec-5-yn-1-ol 36, which was then selectively reduced to (*Z*)-octadec-5-en-1-ol 37 using a Lindlar catalyst, following the procedure previously described for the synthesis of alkene 5. Octadecenol 37 was converted to (Z)-octadec-5-ene-1-thiol 39 and subsequently transformed into methyl ester 40 bearing two C18_D5 tails, as outlined in the procedure used for compound 9. Methyl ester 40 was hydrolyzed to obtain carboxylic acid 41, which was then subjected to amidation with various amines — 28, N,N-dimethyl-2-(piperazin-1-yl)ethan-1-amine, and amine 32 —to synthesize lipids A2C18_D5, A3C18_D5, and A4C18_D5, respectively. For the synthesis of lipid A1C18_D5, methyl ester 40 was directly amidated with N,N-dimethylethane-1,2-diamine using the same method described earlier for compound A1C11_D5.

*Synthesis of A2C18_D9*

Scheme 7. Synthesis scheme of A2C18_D9

Lipid A2C18_D9 was synthesized from methyl ester 14 by its hydrolysis to the carboxylic acid 42, followed by its amidation using amine 28 to give the final lipid.

*Synthesis of A2C18_D9_D12*

Scheme 8. Synthesis scheme of A2C18_D9_D12

Lipid A2C18_D9_D12 was synthesized from methyl ester 19 by its hydrolysis to the carboxylic acid 43, followed by its amidation using the amine 28 to give the final lipid.

NMR spectra and LC-MS data of final products and selected intermediates can be found at the end of the Supporting Information.

Table 1: Overview of lipids tested in the current work.

| Name | Structure |
| --- | --- |
| DLin-MC3-DMA | 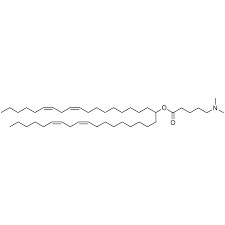 |
| A1C11 |  |
| A1C11_D5 |  |
| A1C18_D9 |  |
| A1C18_D9_D12 |  |
| A1C18_D5 |  |
| A1C18 |  |
| A2C18_D5 |  |
| A3C18_D5 |  |
| A4C18_D5 |  |
| A2C18_D9 |  |
| A2C18_D9_D12 |  |

***Melting temperature (T_m_) analysis of lipids:***

Thermoanalytical measurements of the ionizable lipids were performed on a DSC3+ (Mettler Toledo, Germany). Samples (~10 mg) in sealed aluminum pans were heated from -80 to 100 °C under a nitrogen atmosphere, using a heating rate of 10 K/min. The onset of the phase transition temperature (T_m_) is reported in °C and shown in Figure 2B.

## Biological testing of LNPs containing novel thioether lipids.


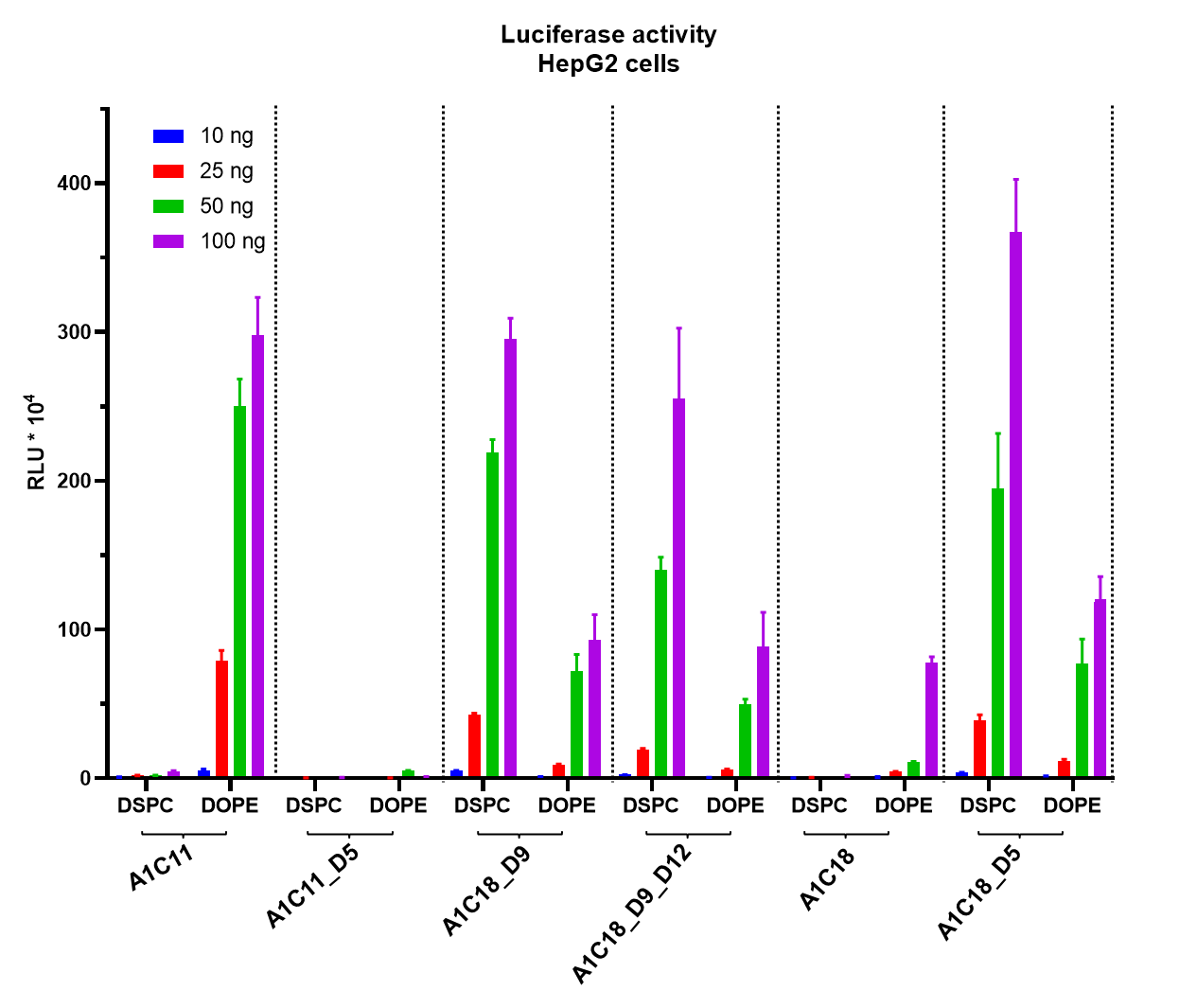


Figure 1: HepG2 cells were treated with LNPs containing mRNA encoding for Luciferase (at four different mRNA doses) for 24 h and the luciferase activity was determined after 24 h. Selected data was extracted from this graph and is shown in Figure 1. Mean values from triplicates are shown ± standard deviations.


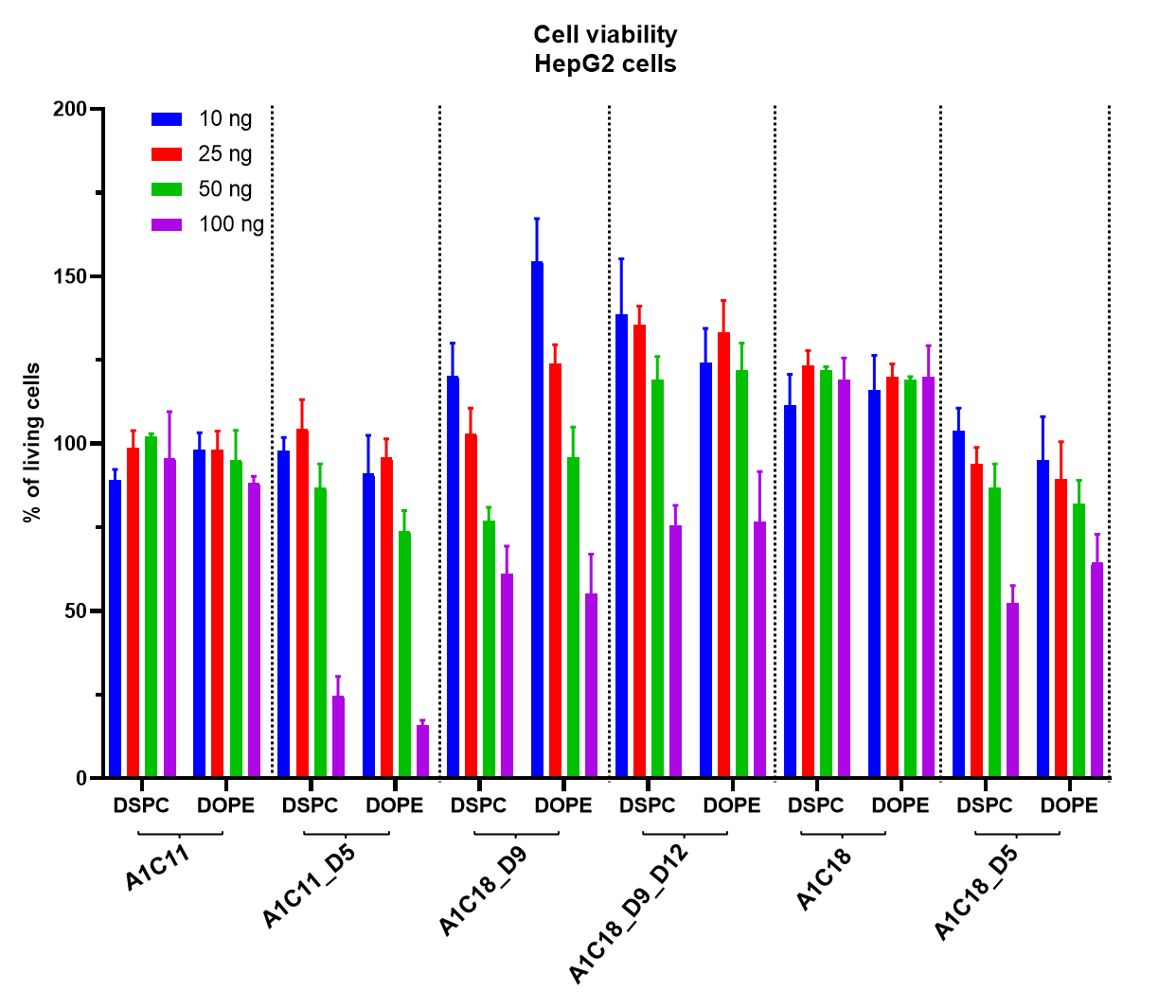


Figure 2: HepG2 cells were treated with LNPs containing mRNA encoding for Luciferase (at four different mRNA doses) for 24 h and the cell viability was determined afterwards. Untreated cells were used as control and the cell viability was set to 100%. Mean values from triplicates are shown ± standard deviations.


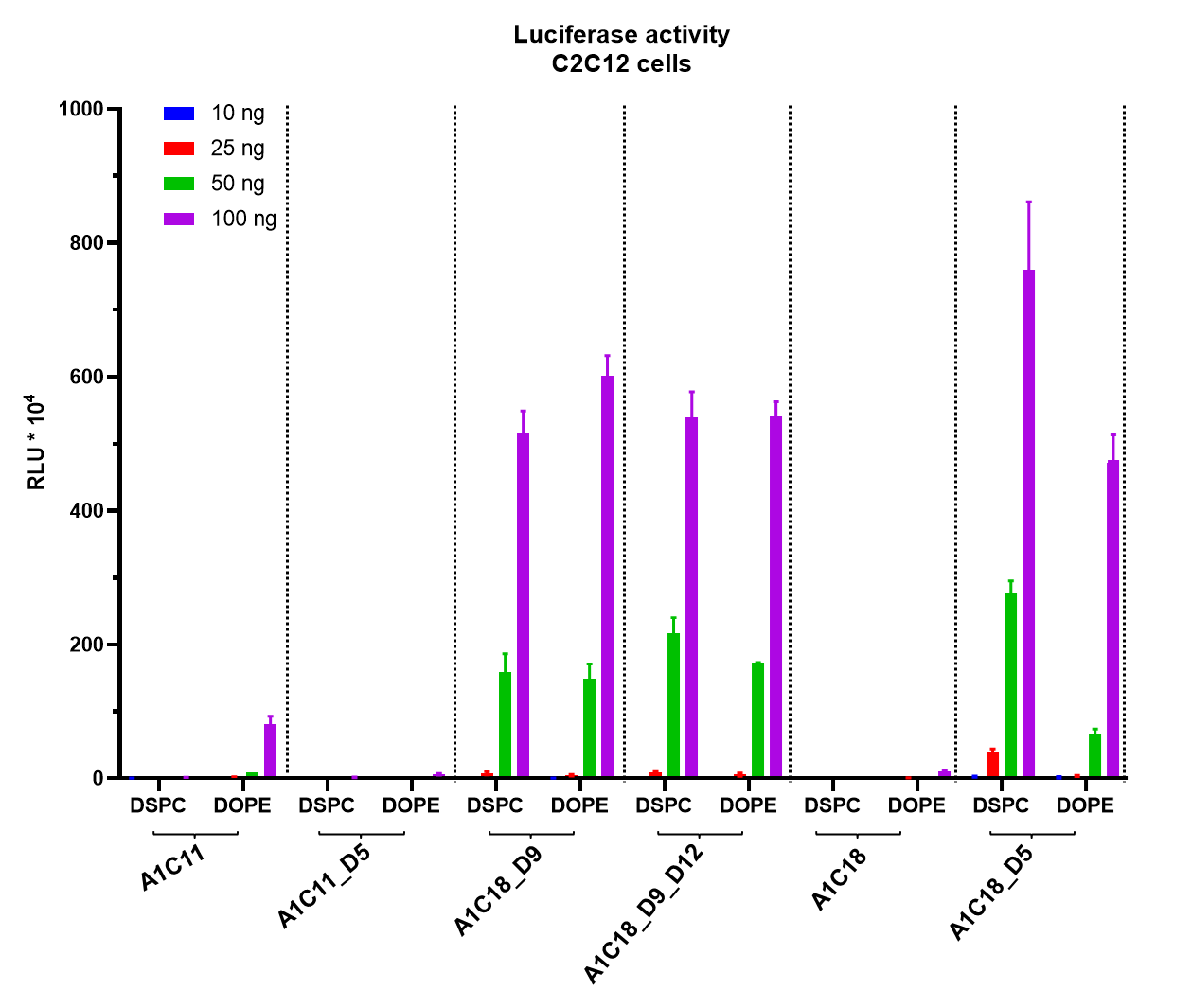


Figure 3: C2C12 cells were treated with LNPs containing mRNA encoding for Luciferase (at four different doses 10 – 100 ng) for 24 h and the luciferase activity was determined afterwards. Selected data was extracted from this graph and is shown in Figure 1. Mean values from triplicates are shown ± standard deviations.


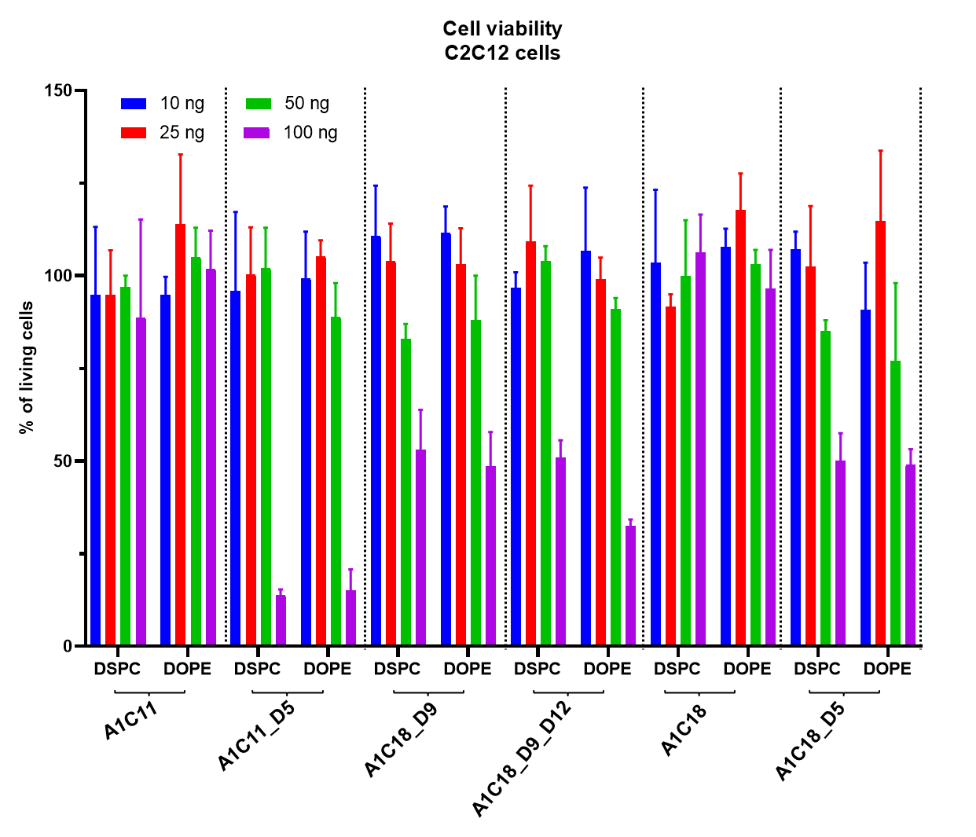


Figure 4: C2C12 cells were treated with LNPs containing mRNA encoding for Luciferase (at four different doses 10 – 100 ng) for 24 h and the cell viability was determined afterwards. Untreated cells were used as control and the cell viability was set to 100%. Mean values from triplicates are shown ± standard deviations.


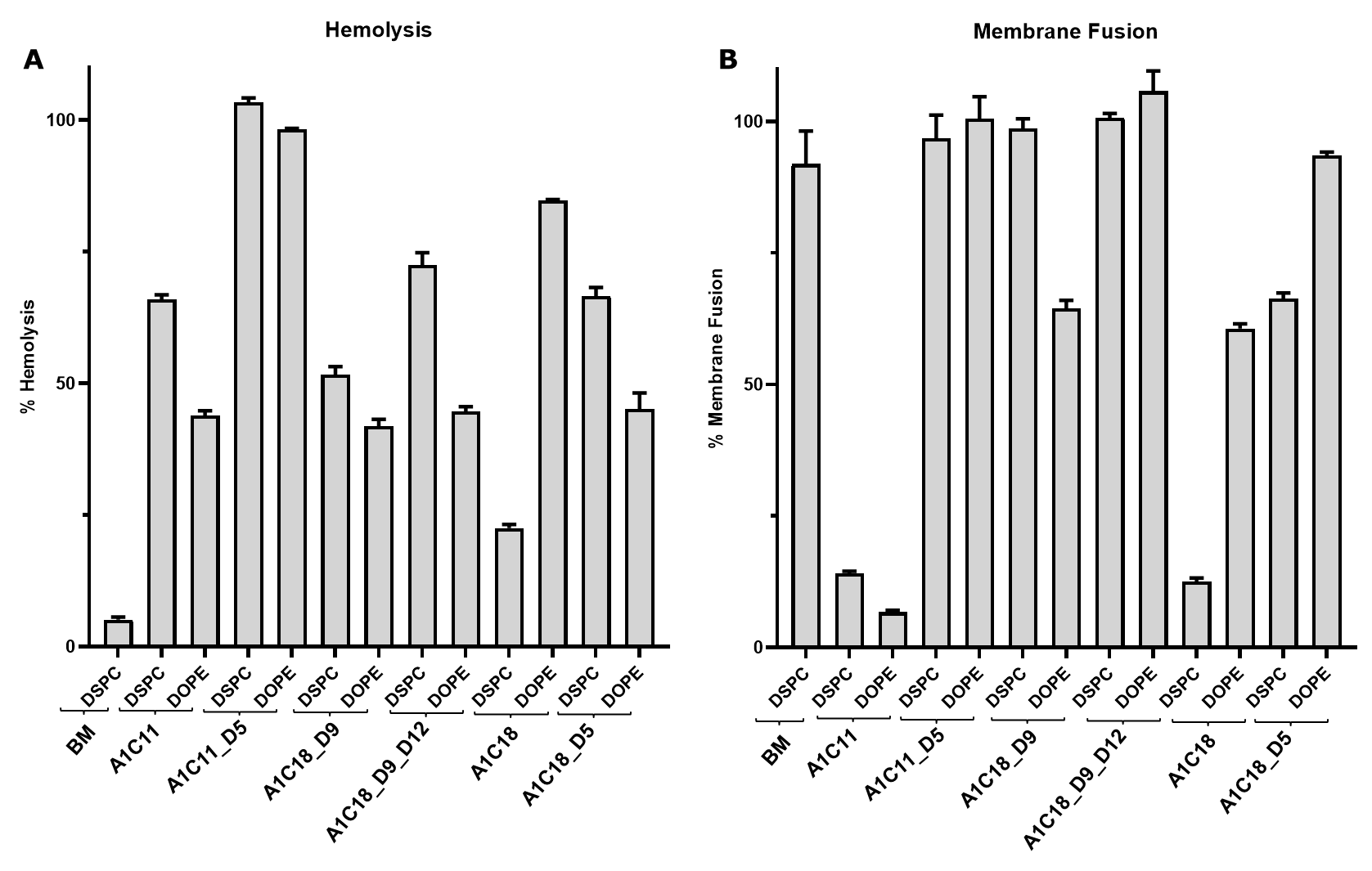


Figure 5: Red blood cells were incubated in PBS at pH = 7.4 (A) or in buffer at pH = 5.5 with LNPs for 1 h. The absorption was measured afterwards at 540 nm. Selected data was extracted from this graph and is shown in Figure 2. Mean values from duplicates are shown ± standard deviations.


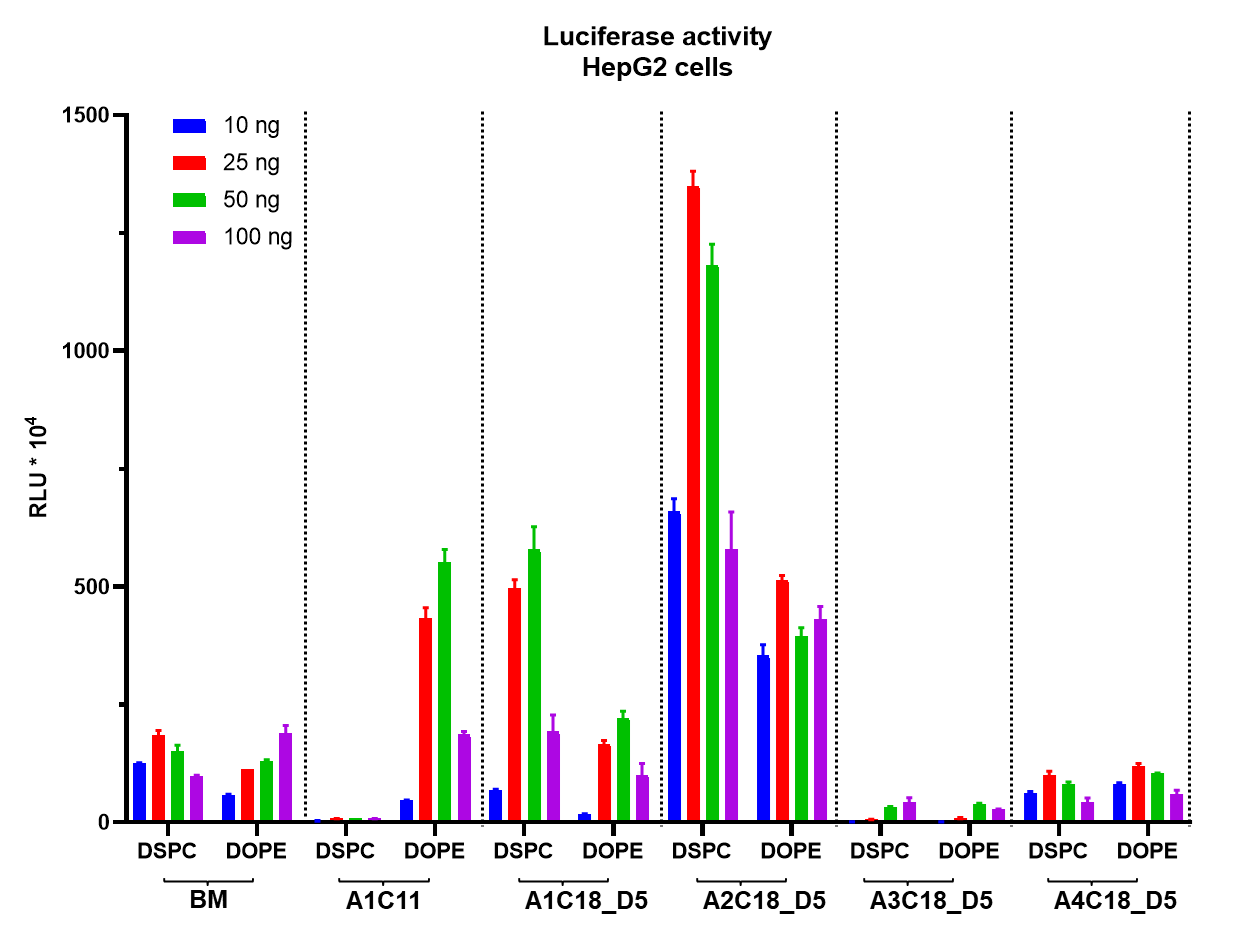


Figure 6: HepG2 cells were treated with LNPs containing mRNA encoding for Luciferase (at four different doses 10 – 100 ng) for 24 h and the luciferase activity was determined afterwards. Mean values from triplicates are shown ± standard deviations. BM = Benchmark LNP


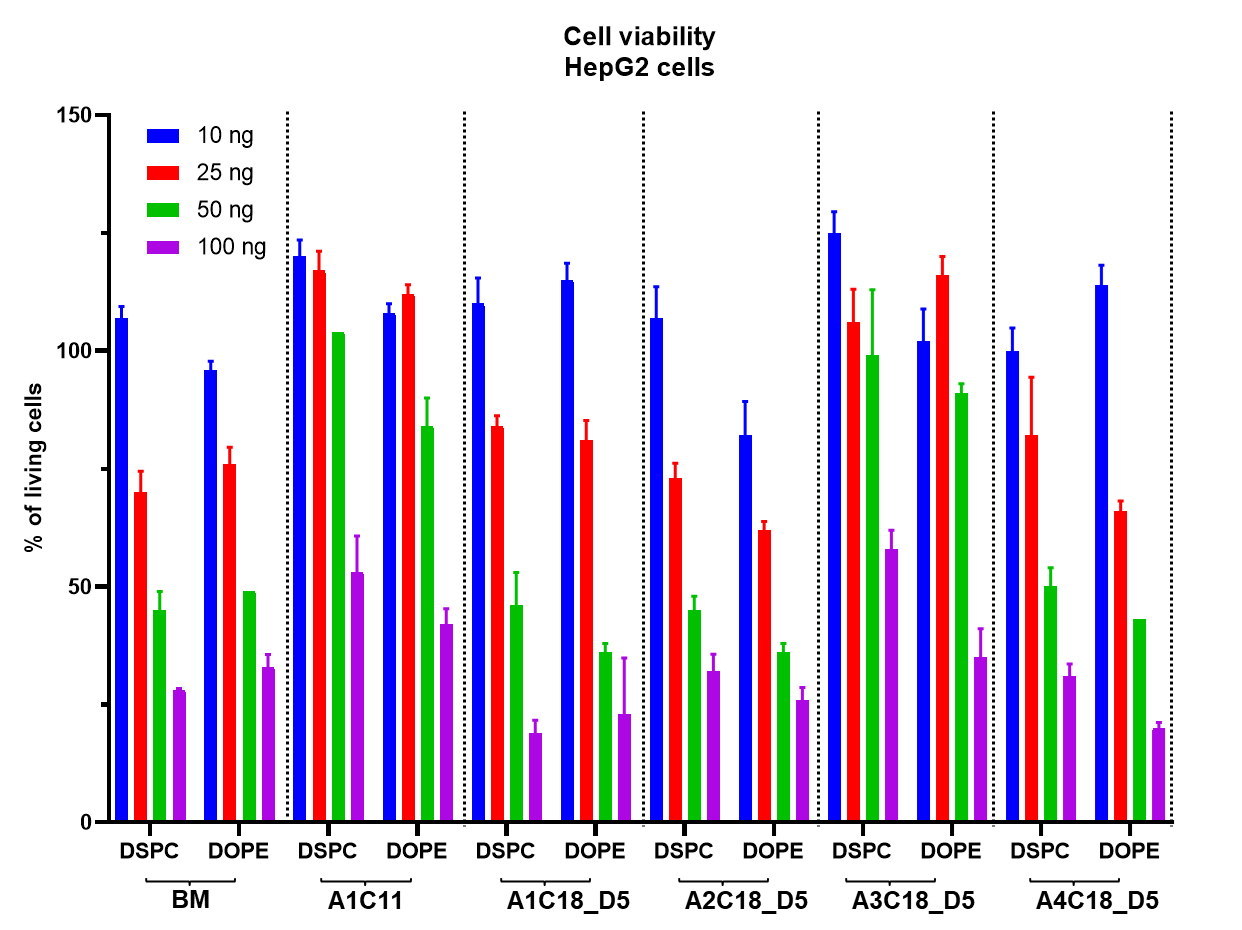


Figure 7: HepG2 cells were treated with LNPs containing mRNA encoding for Luciferase (at four different doses 10 – 100 ng) for 24 h and the cell viability was determined afterwards. Untreated cells were used as control and the cell viability was set to 100%. Mean values from triplicates are shown ± standard deviations. BM = Benchmark LNP


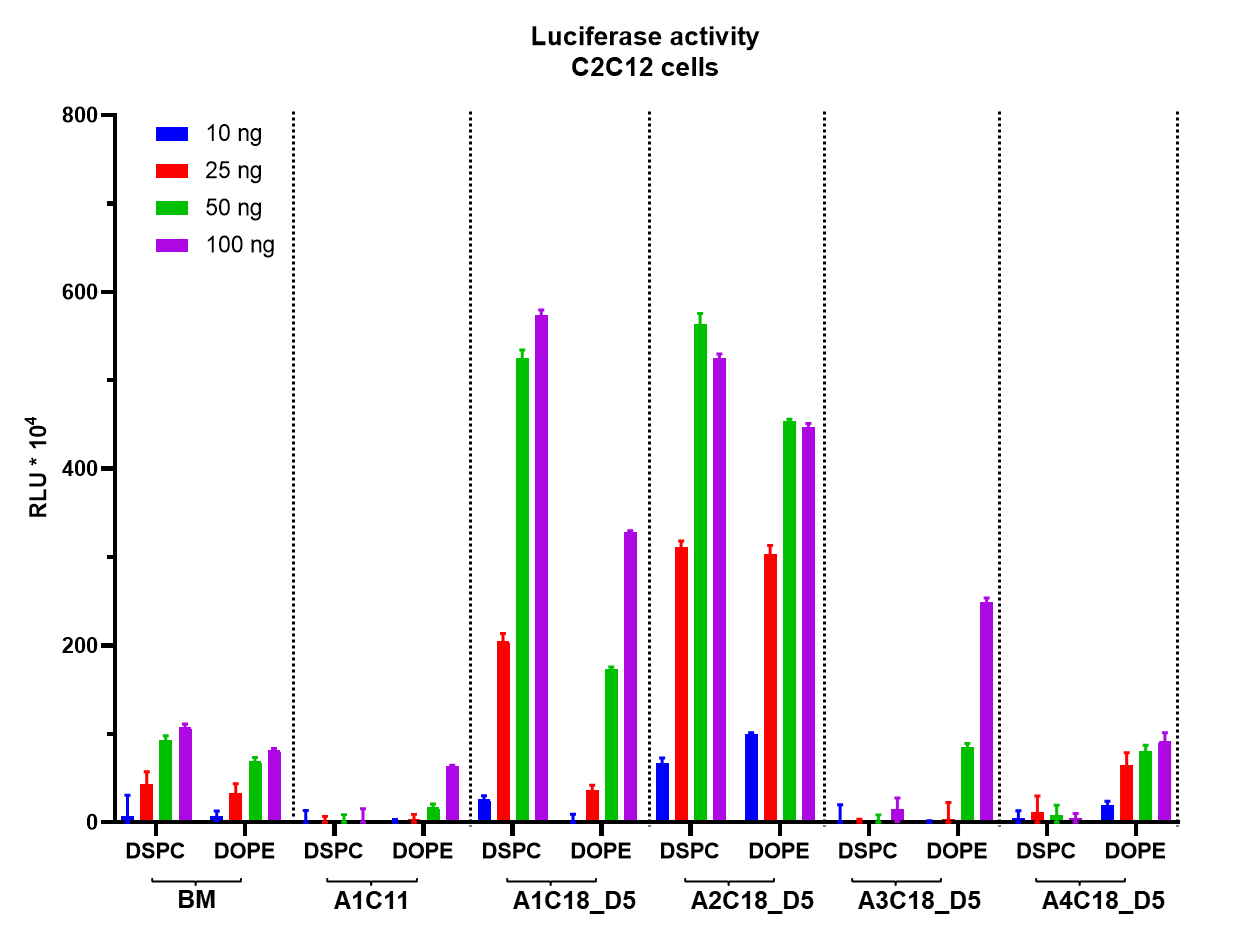


Figure 8: C2C12 cells were treated with LNPs containing mRNA encoding for Luciferase (at four different doses 10 – 100 ng) for 24 h and the luciferase activity was determined afterwards. Mean values from triplicates are shown ± standard deviations. BM = Benchmark LNP


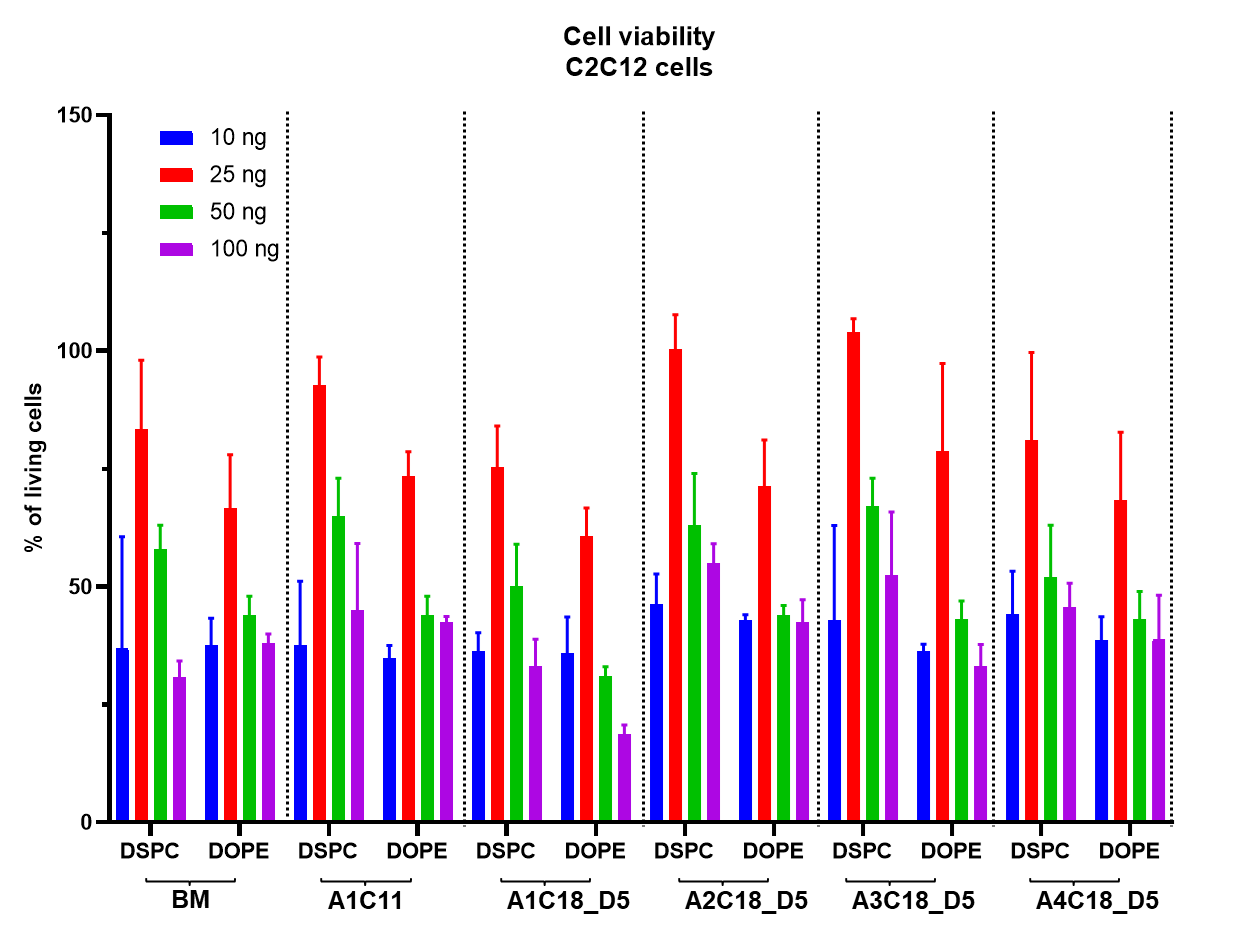


Figure 9: C2C12 cells were treated with LNPs containing mRNA encoding for Luciferase (at four different doses 10 – 100 ng) for 24 h and the cell viability was determined afterwards. Untreated cells were used as control and the cell viability was set to 100%. Mean values from triplicates are shown ± standard deviations. BM = Benchmark LNP

Figure 10: HepG2 cells were treated with LNPs containing mRNA encoding for Luciferase (at four different doses 10 – 100 ng) for 24 h and the cell viability was determined afterwards. Untreated cells were used as control and the cell viability was set to 100%. Selected data was extracted from this graph and is shown in Figure 3. Mean values from triplicates are shown ± standard deviations.

Figure 11: HepG2 cells were treated with LNPs containing mRNA encoding for Luciferase (at four different doses 10 – 100 ng) for 24 h and the cell viability was determined afterwards. Untreated cells were used as control and the cell viability was set to 100%. Mean values from triplicates are shown ± standard deviations.

Figure 12: C2C12 cells were treated with LNPs containing mRNA encoding for Luciferase (at four different doses 10 – 100 ng) for 24 h and the luciferase activity was determined afterwards. Mean values from triplicates are shown ± standard deviations.

Figure 13: C2C12 cells were treated with LNPs containing mRNA encoding for Luciferase (at four different doses 10 – 100 ng) for 24 h and the cell viability was determined afterwards. Untreated cells were used as control and the cell viability was set to 100%. Mean values from triplicates are shown ± standard deviations.


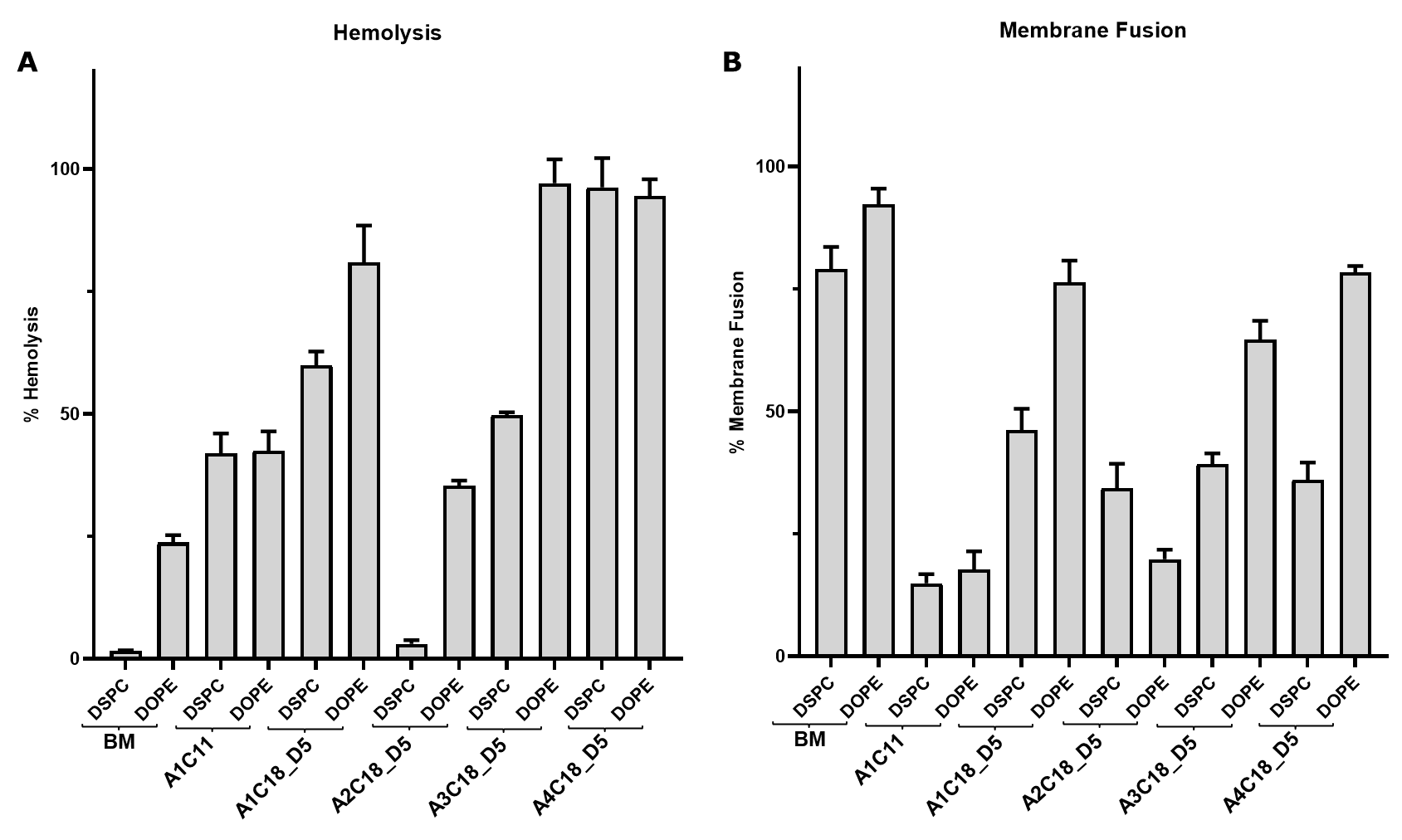


Figure 14: Red blood cells were incubated in PBS at pH = 7.4 (A) or in buffer at pH = 5.5 with LNPs for 1 h. The absorption was measured afterwards at 540 nm. Selected data was extracted from this graph and is shown in Figure 3. Mean values from duplicates are shown ± standard deviations.


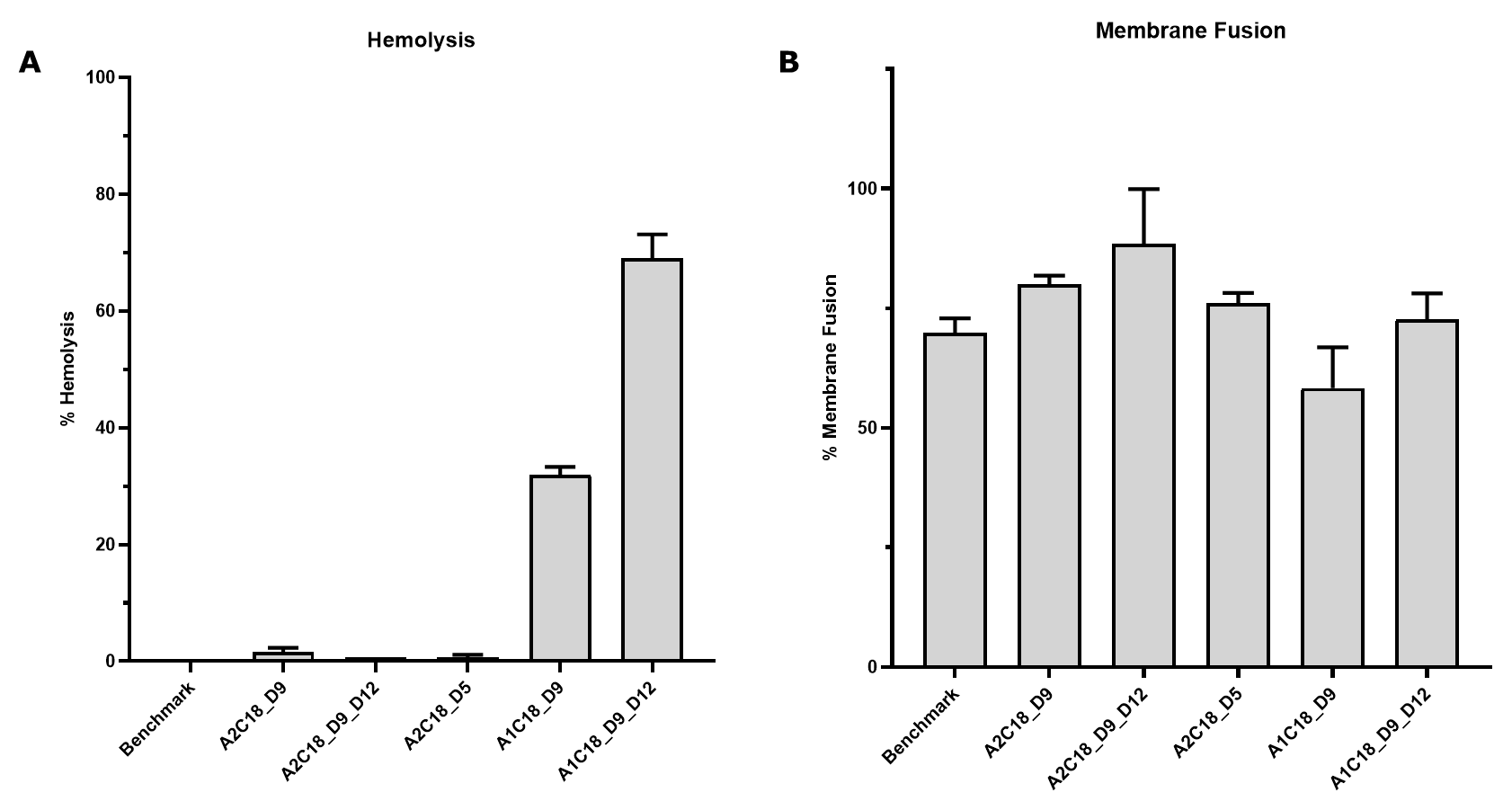


Figure 15: Red blood cells were incubated in PBS at pH = 7.4 (A) or in buffer at pH = 5.5 with LNPs for 1 h. The absorption was measured afterwards at 540 nm. Mean values from duplicates are shown ± standard deviations.


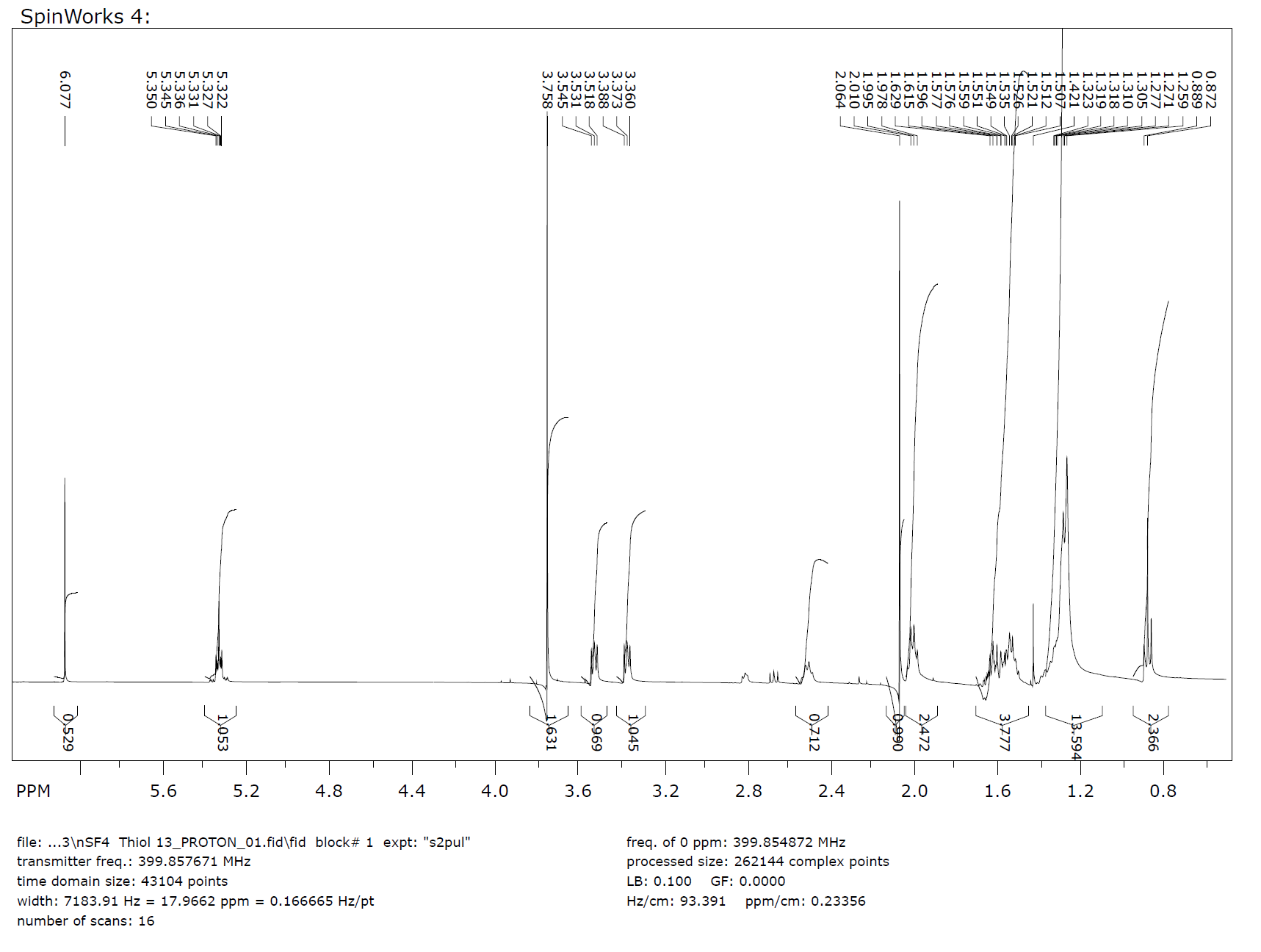


Figure 16: 1H-NMR of compound 13.


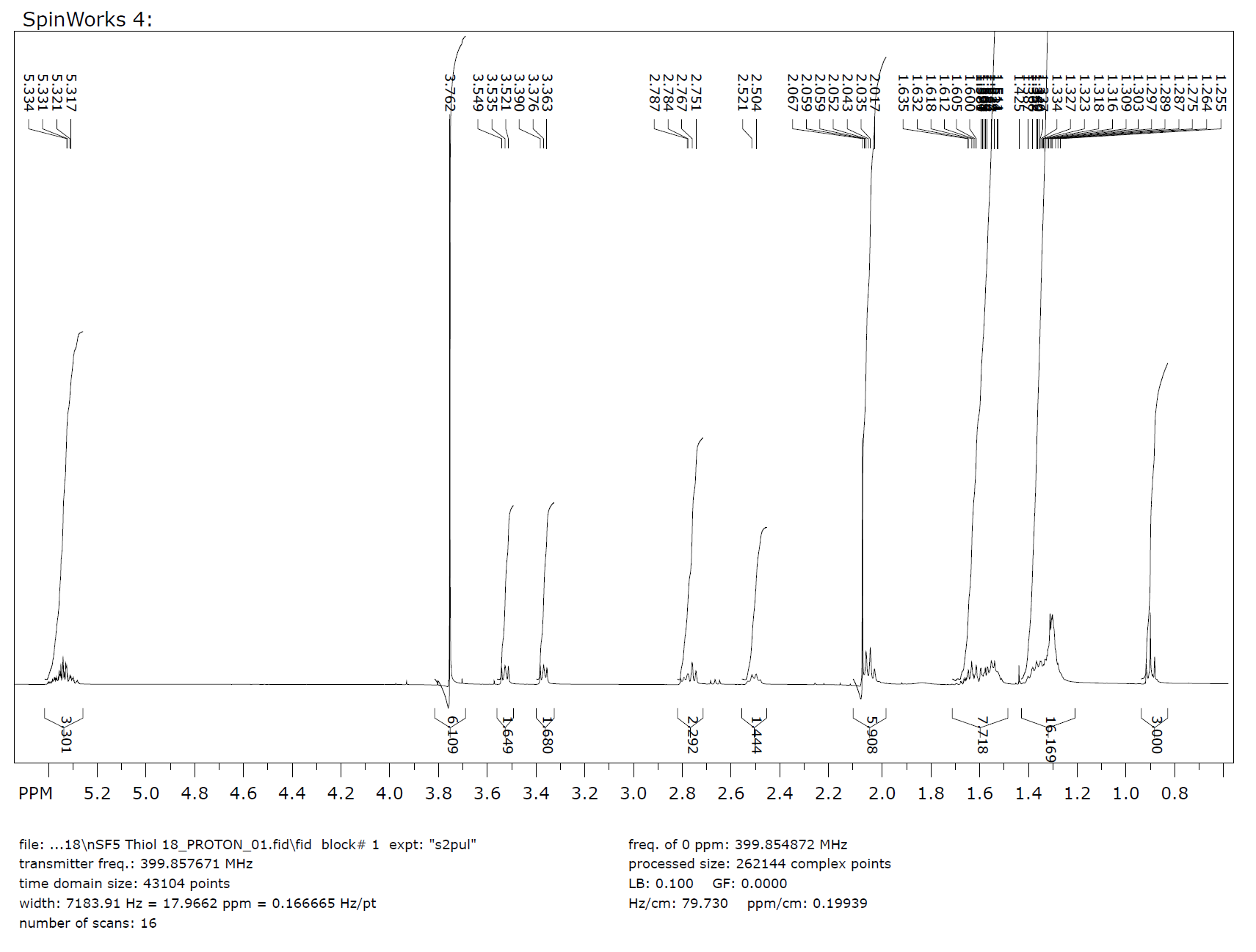


Figure 17: 1H-NMR of compound 18.


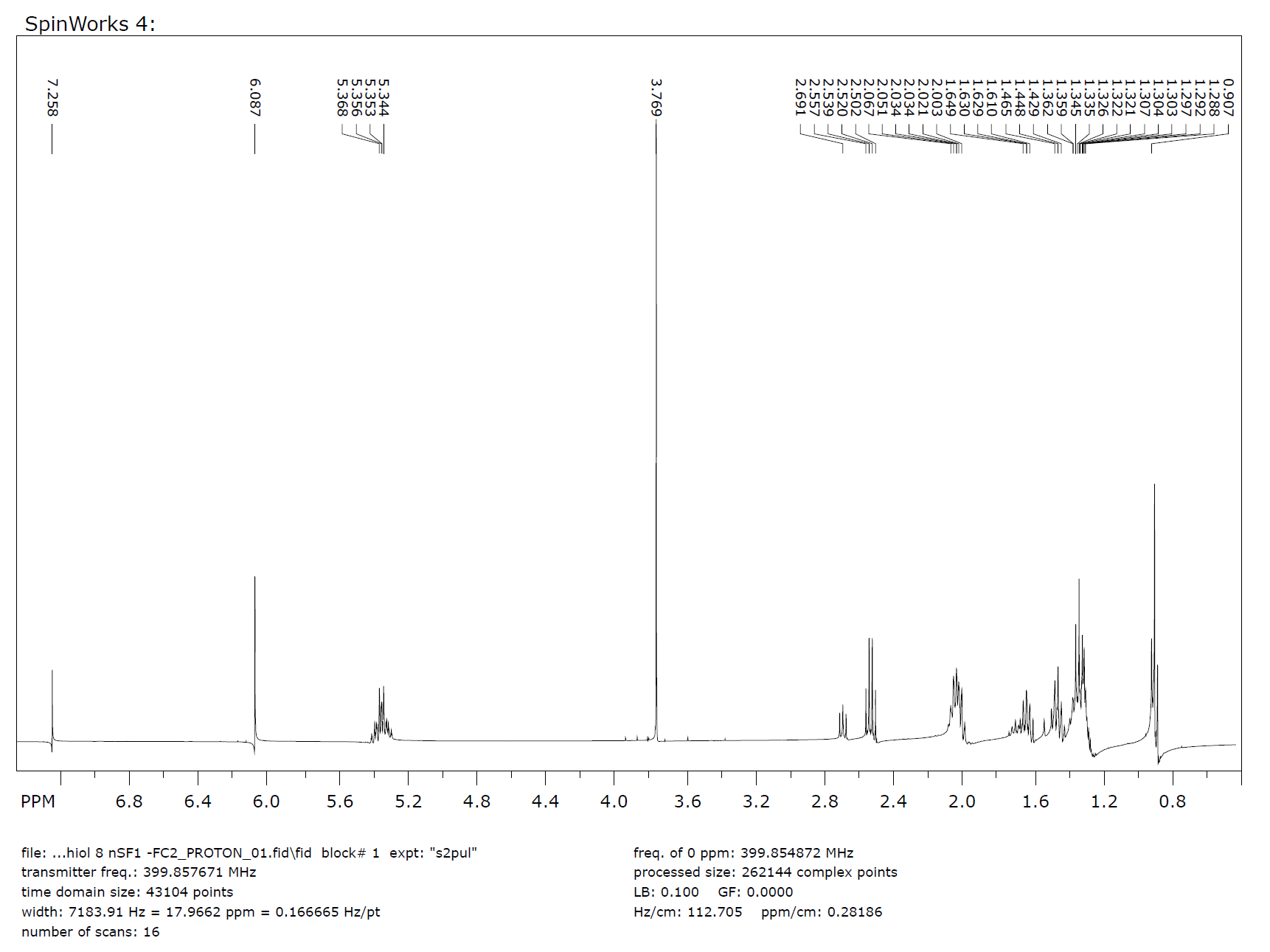


Figure 18: 1H-NMR of compound 8.


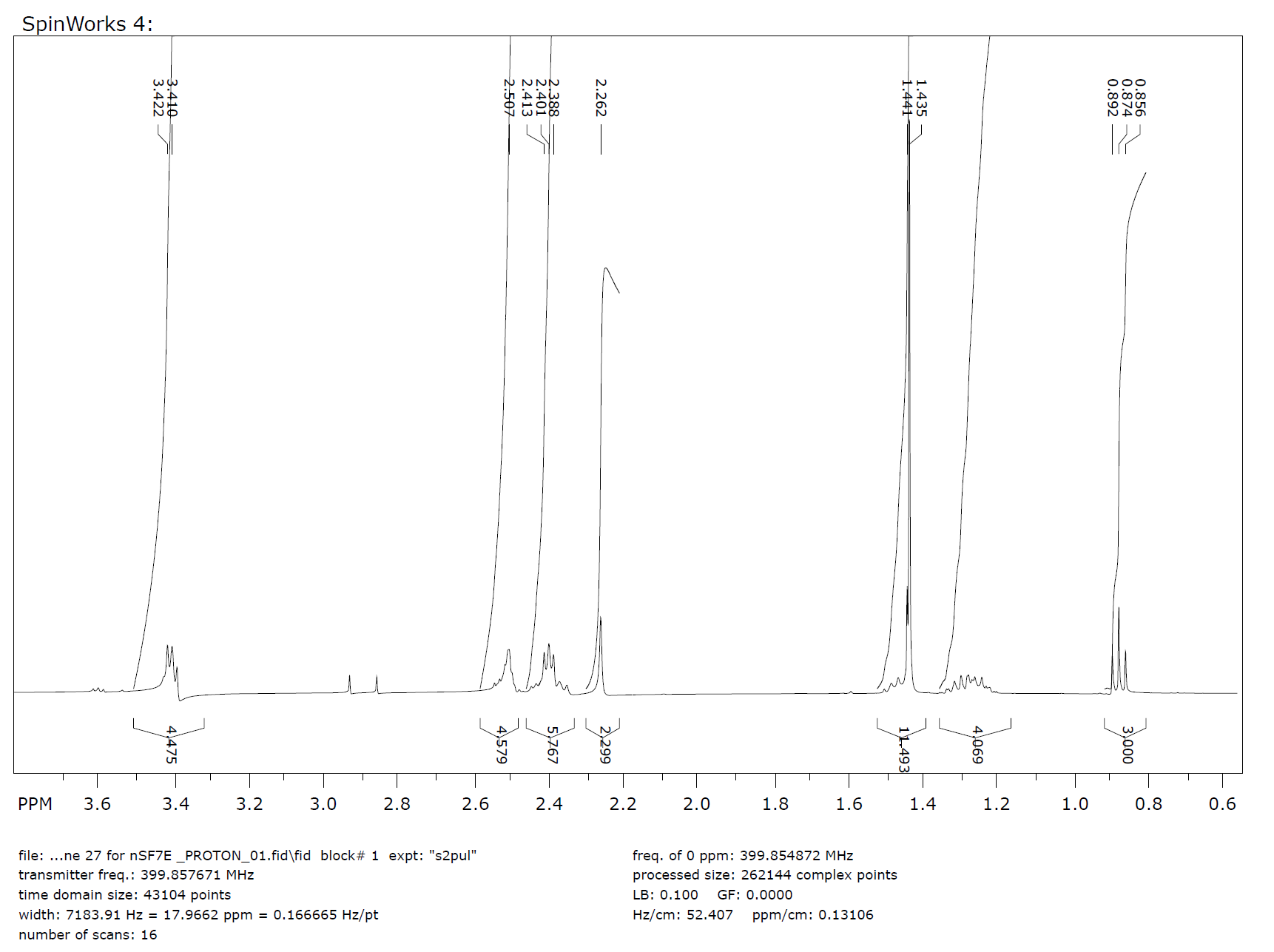


Figure 19: 1H-NMR of compound 27.


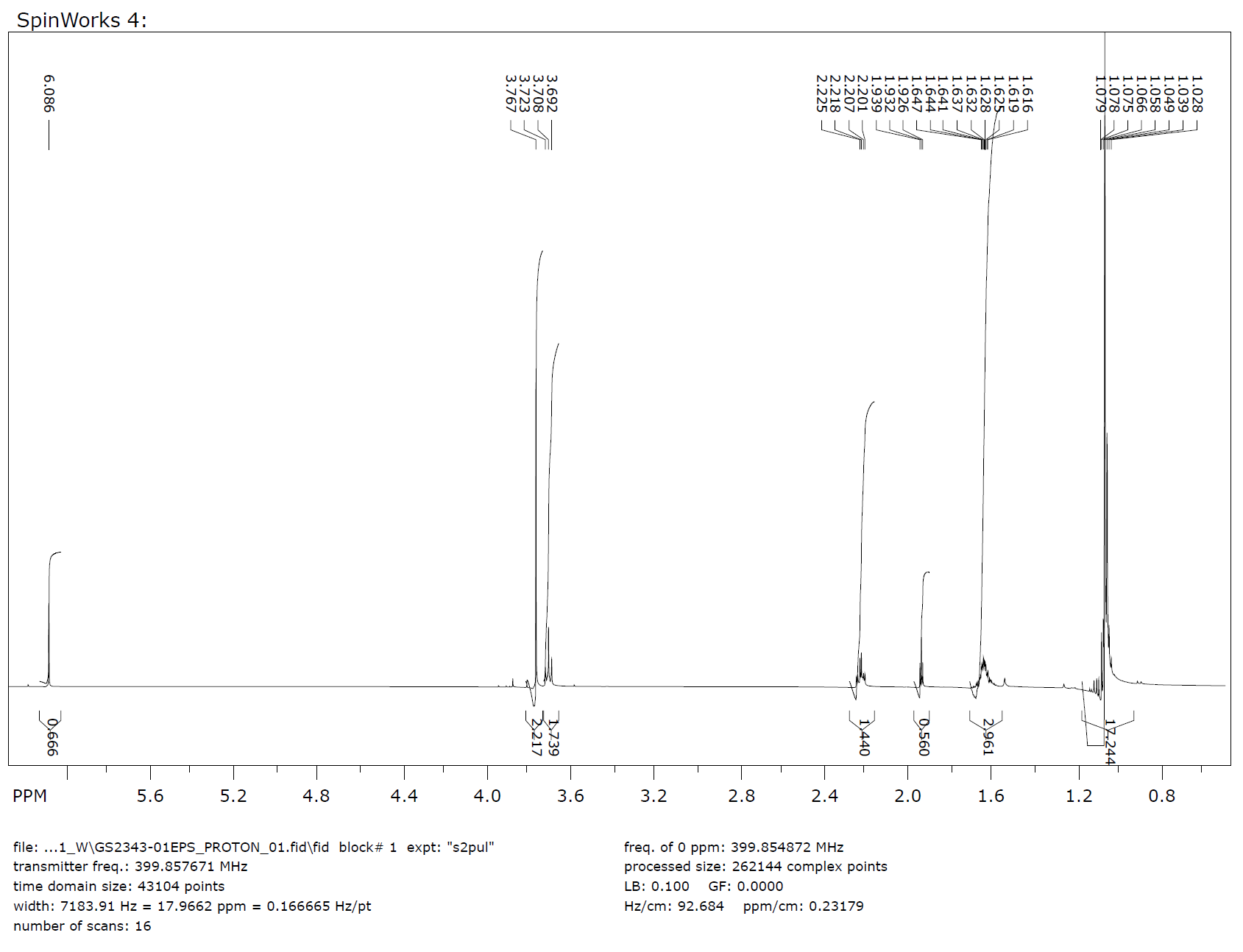


Figure 21: 1H-NMR of compound 34.


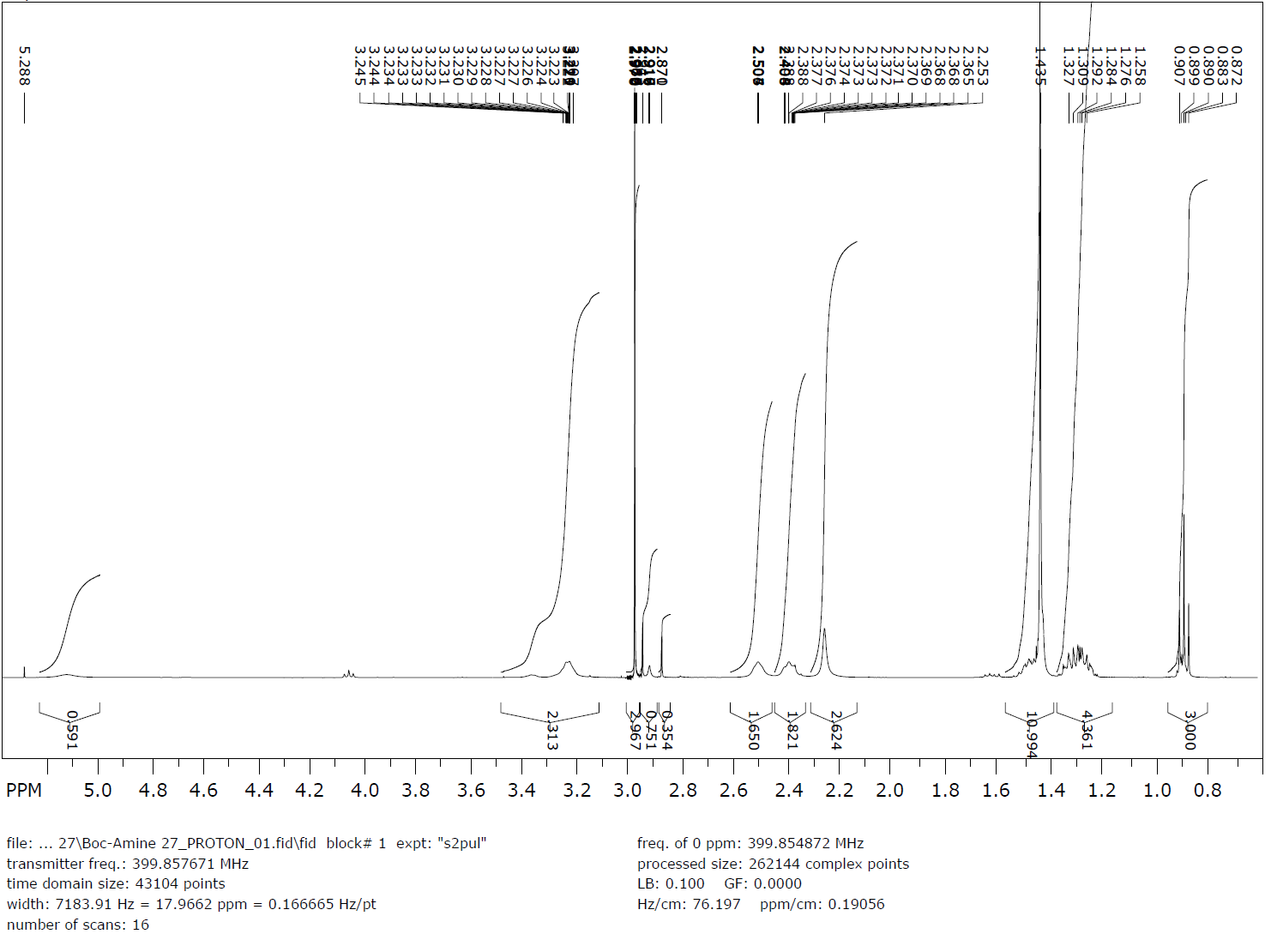


Figure 22: 1H-NMR of compound 27.


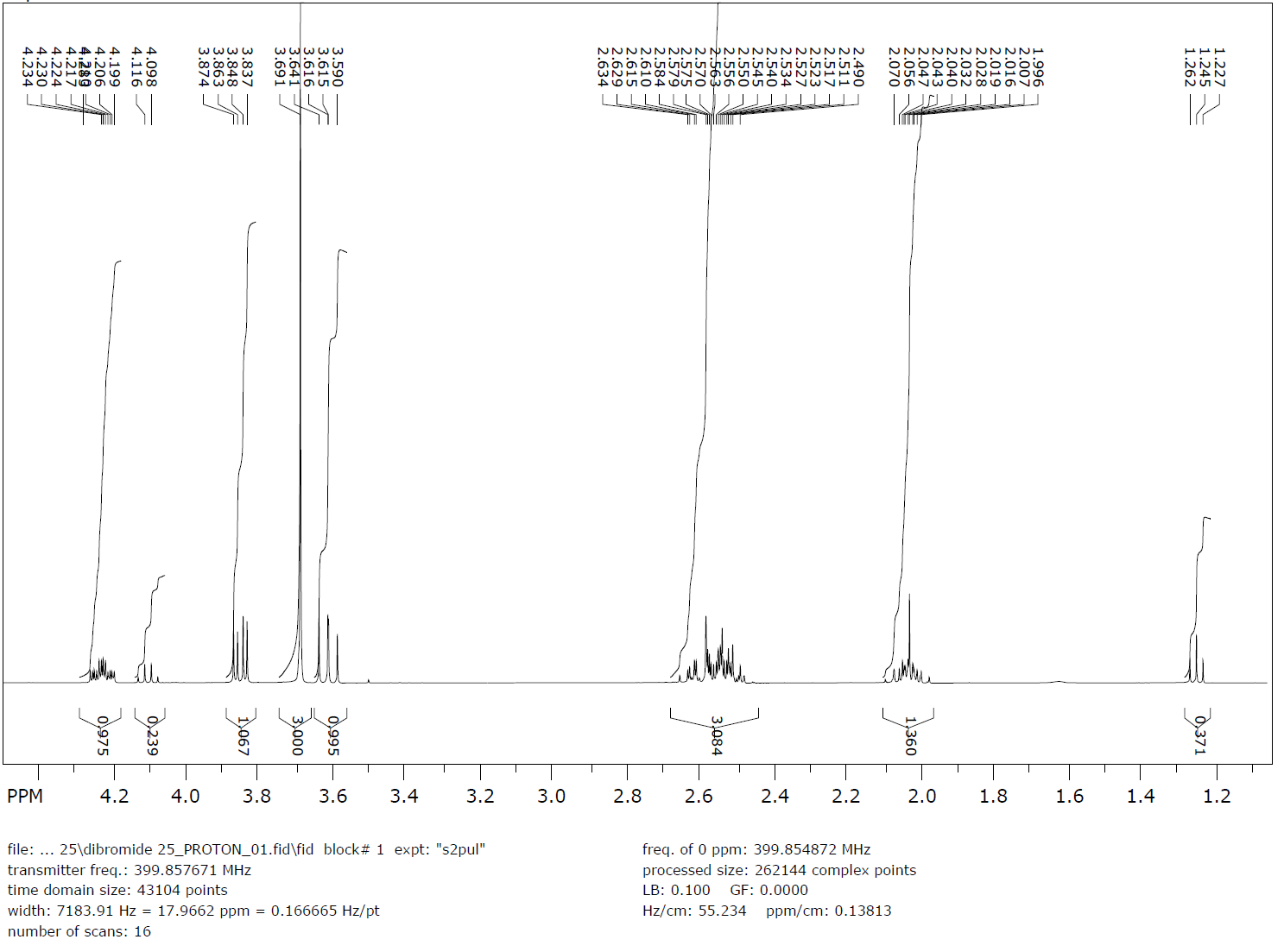


Figure 23: 1H-NMR of compound 25.


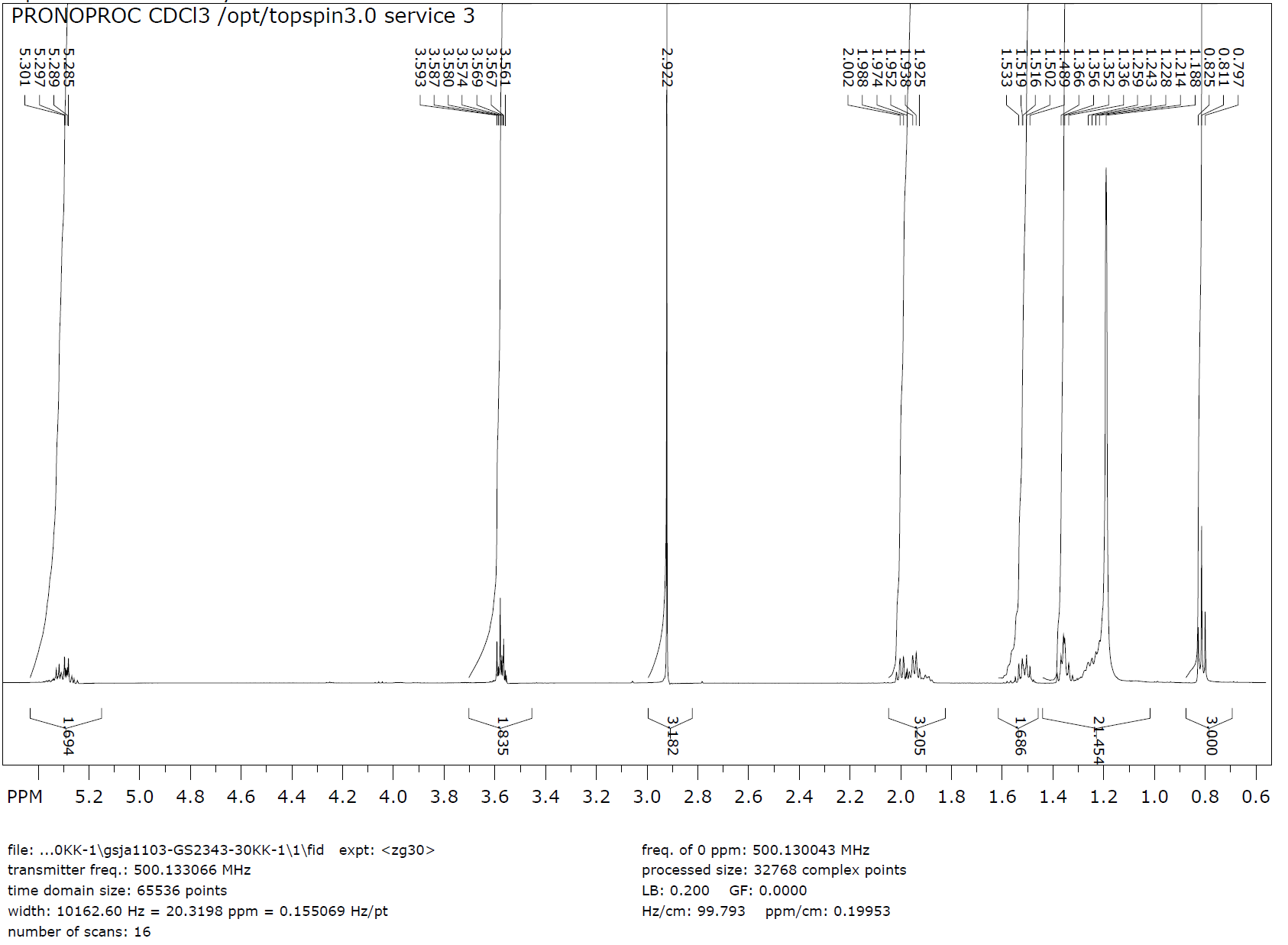


Figure 24: 1H-NMR of compound 37.


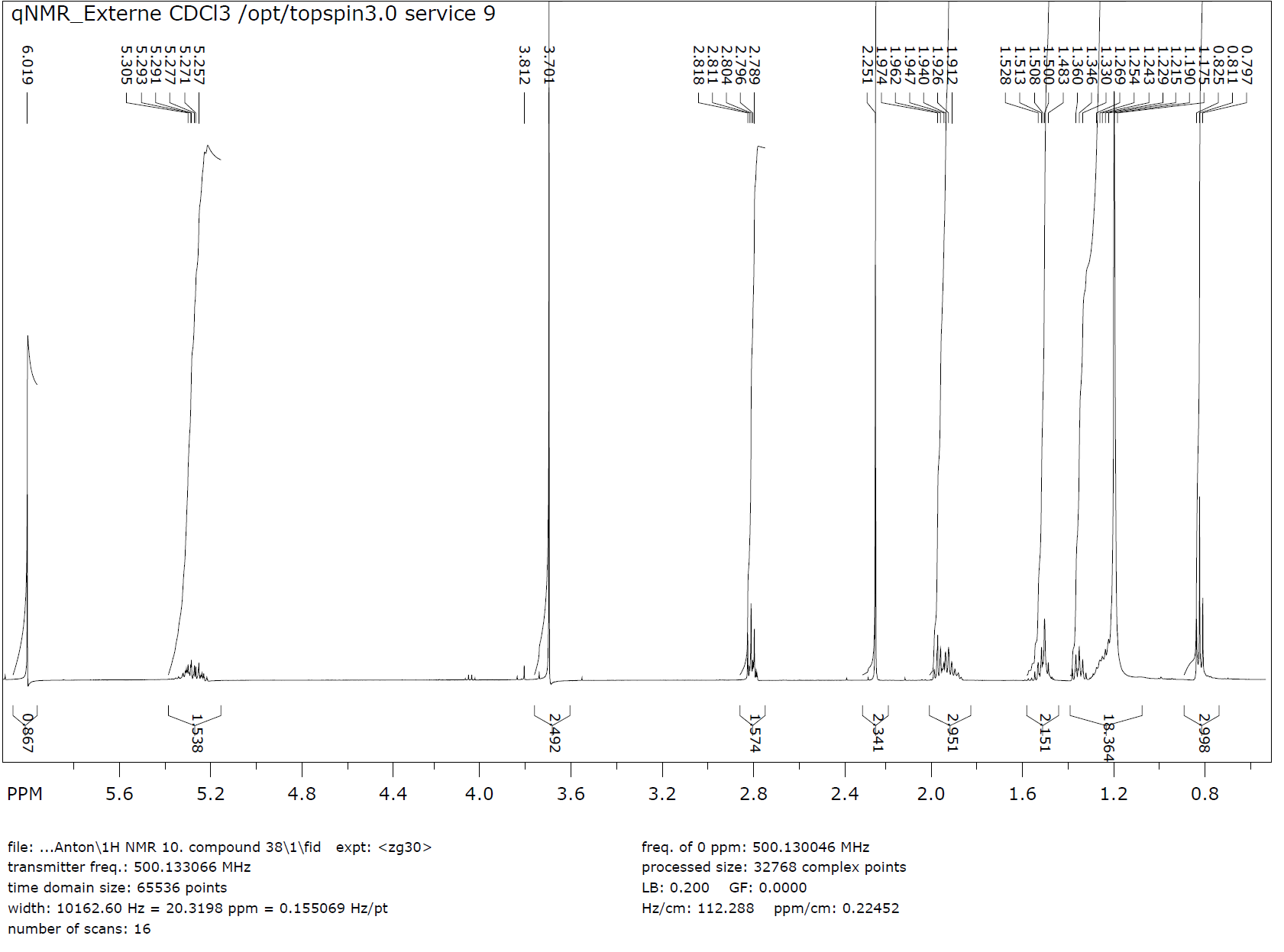


Figure 25: 1H-NMR of compound 38.


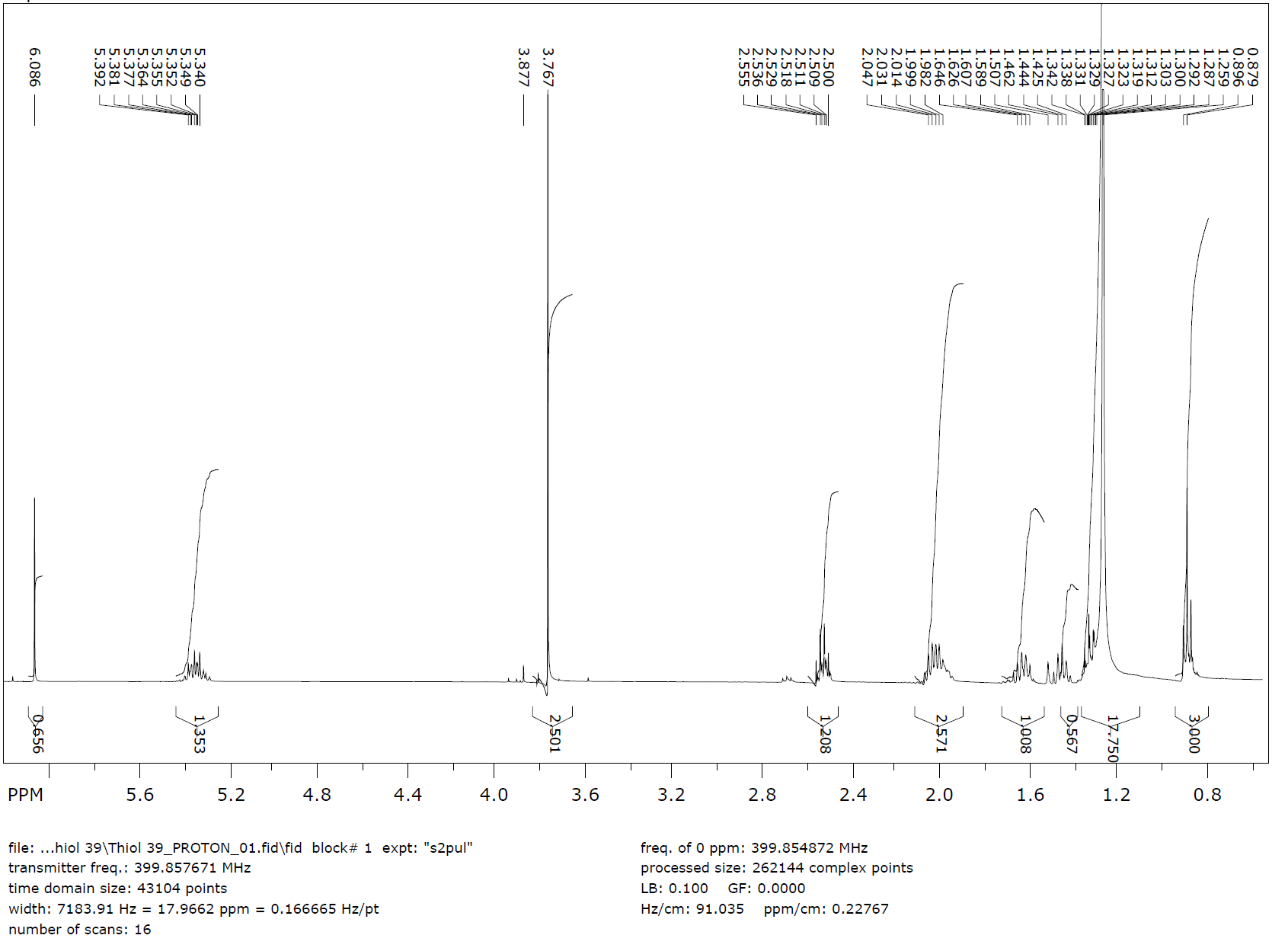


Figure 26: 1H-NMR of compound 39.


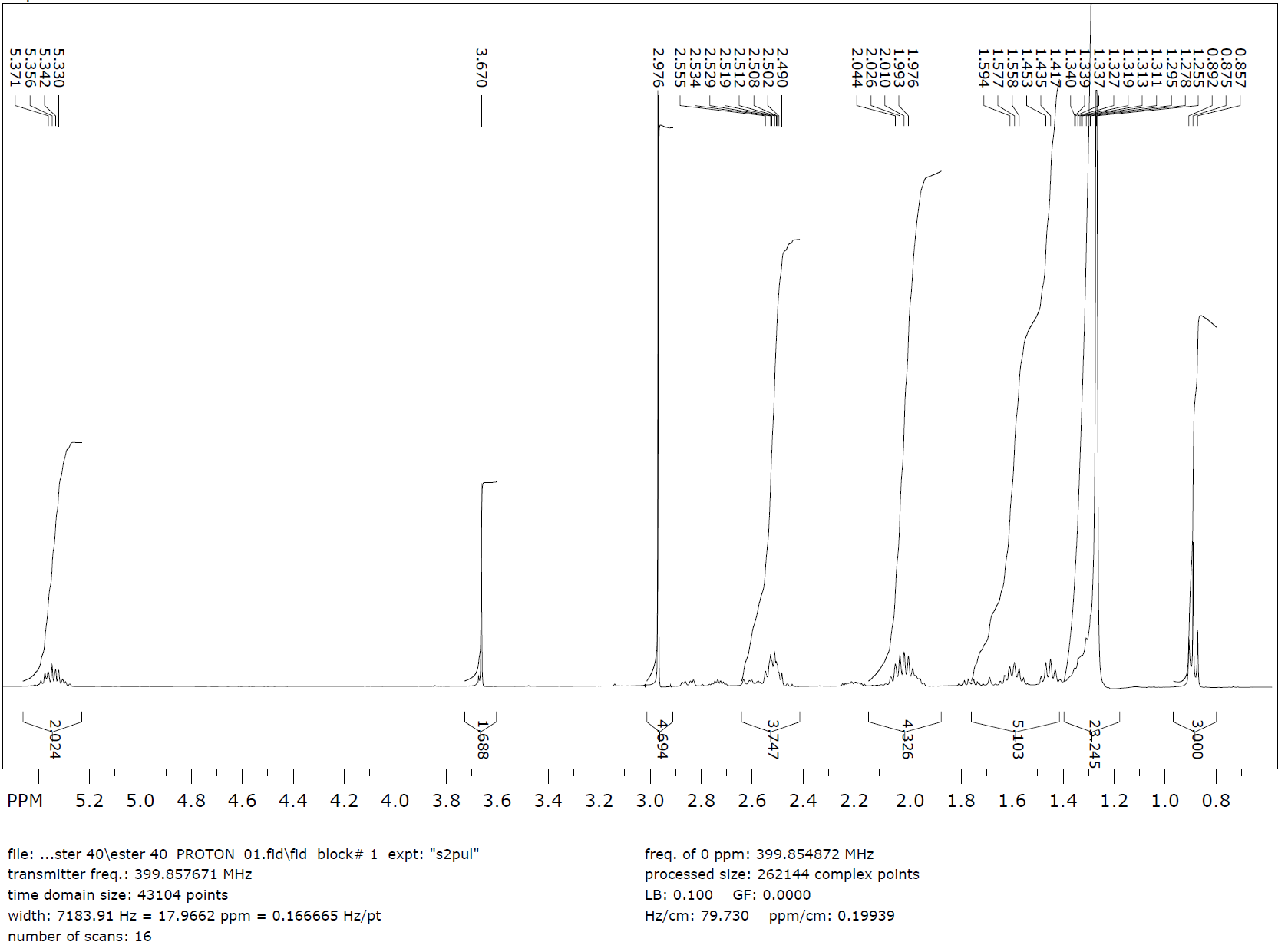


Figure 27: 1H-NMR of compound 40.


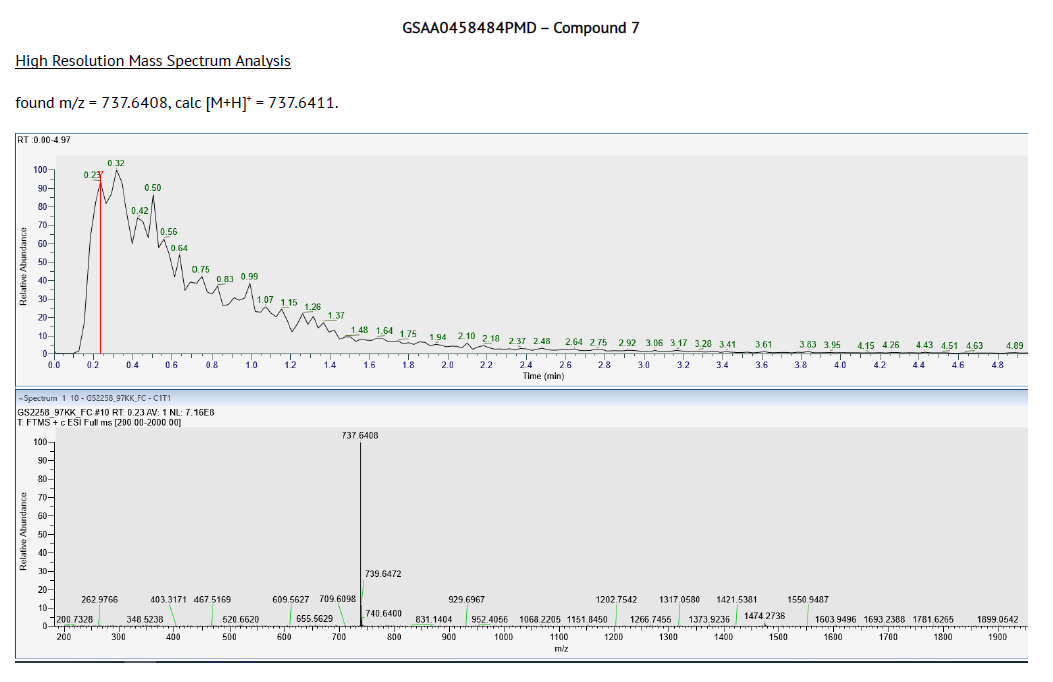


Figure 28: High-resolution MS of compound A1C18_D5


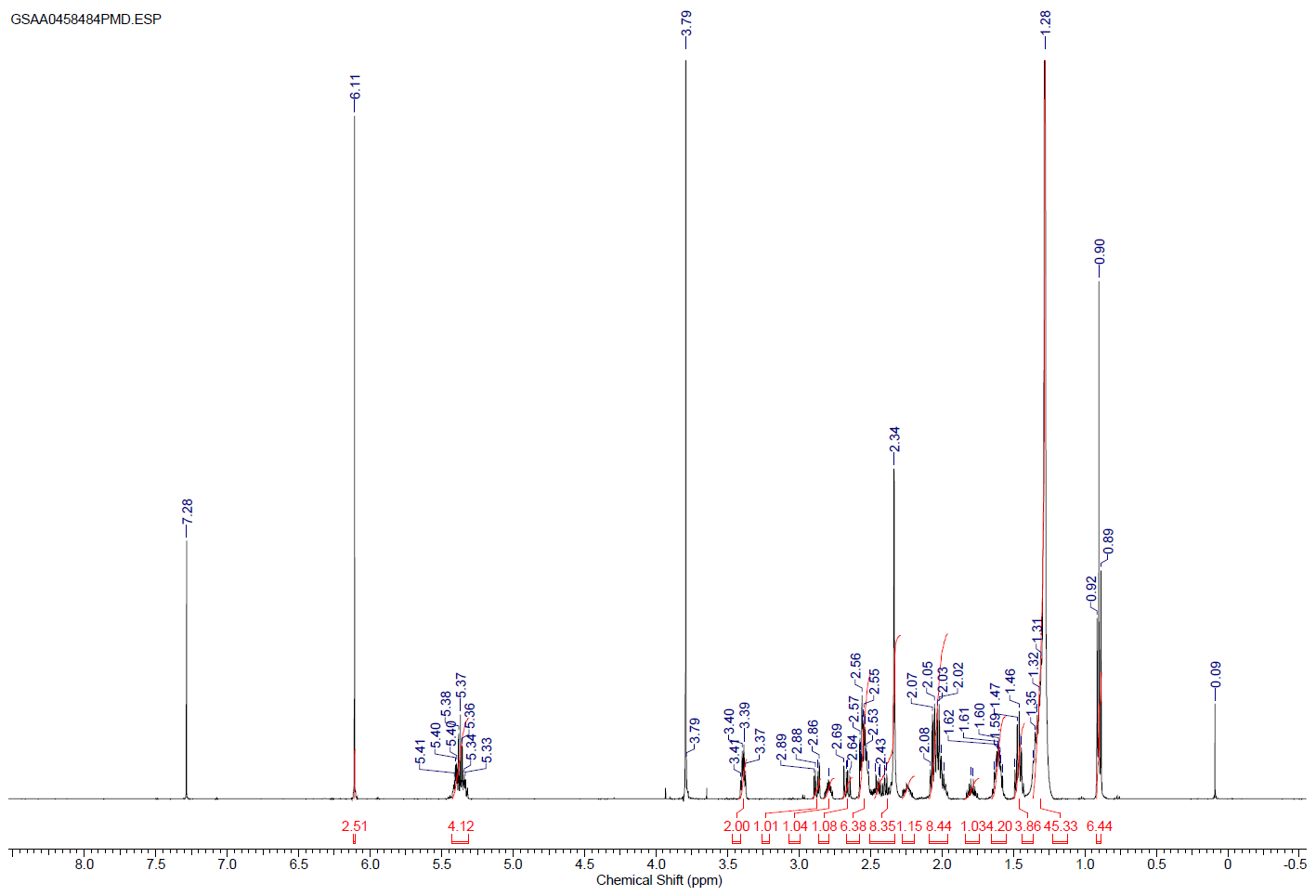


Figure 29: 1H-NMR of A1C18_D5


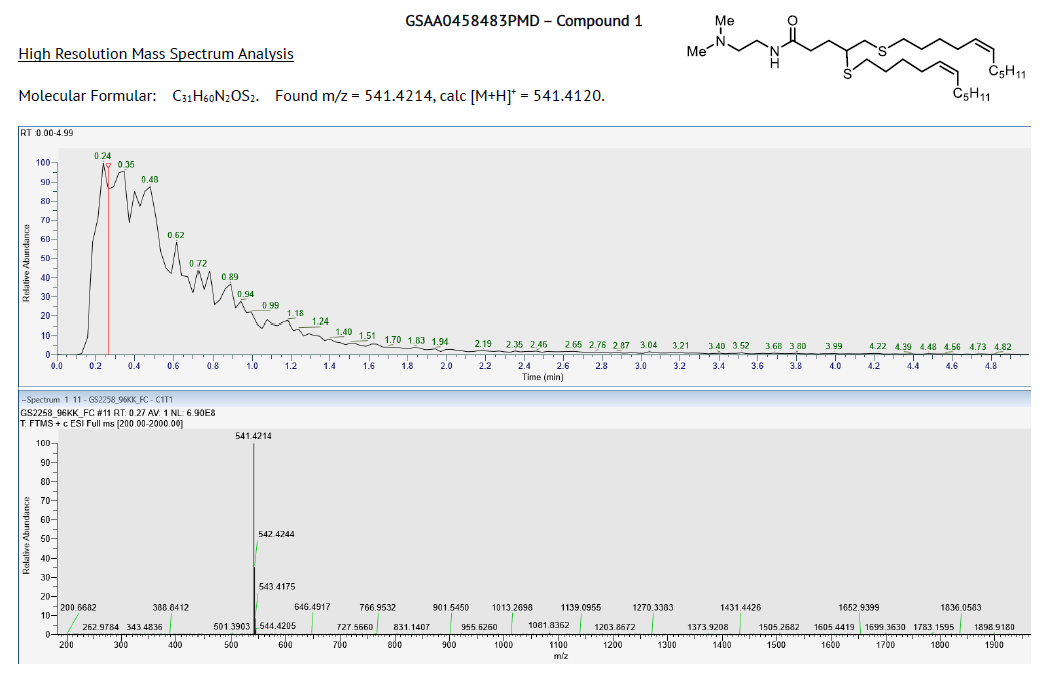


Figure 30: High resolution MS of compound A1C11_D5 (calc. [M+H]+ = 541,4120)


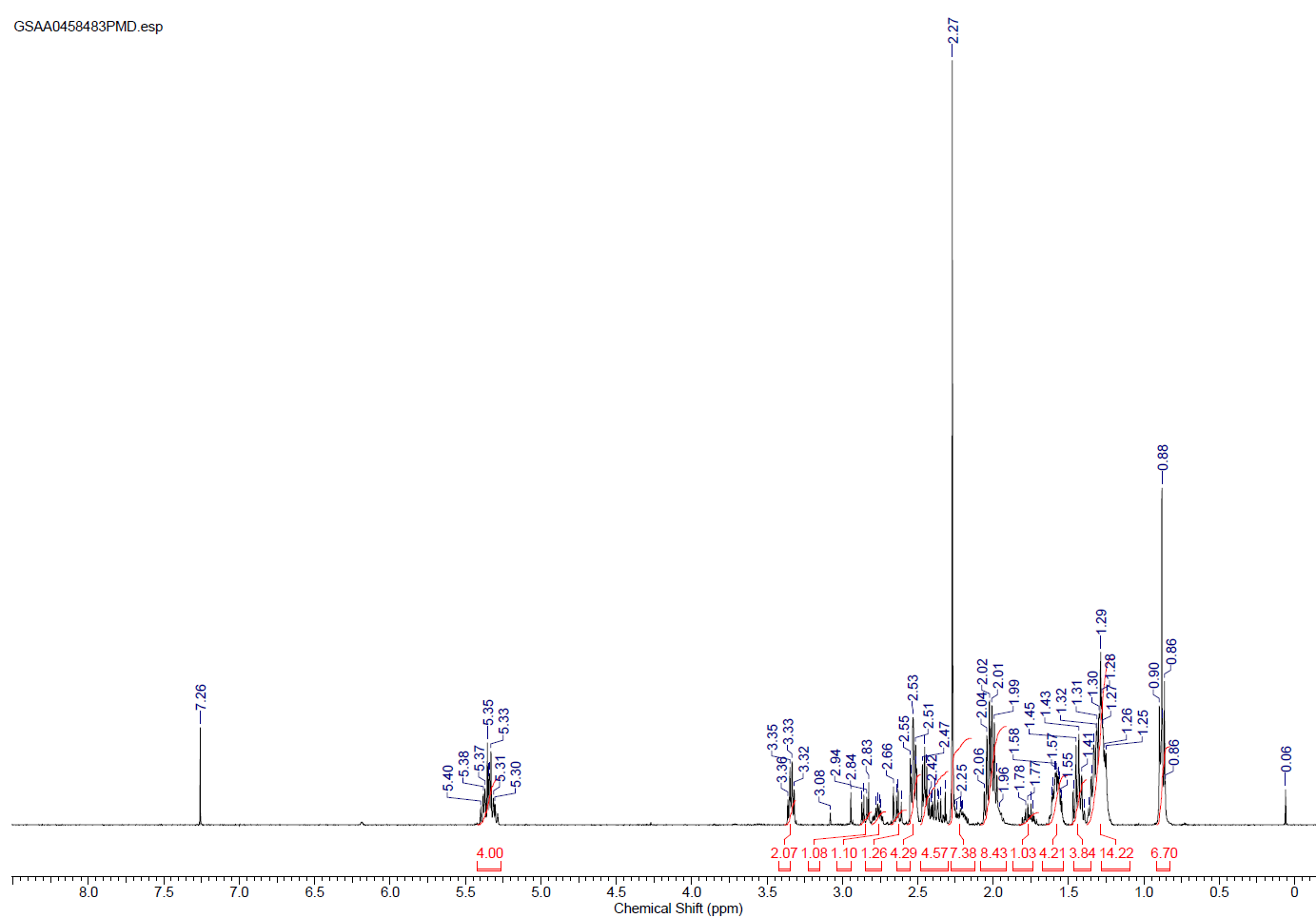


Figure 31: 1H-NMR of compound A1C11_D5


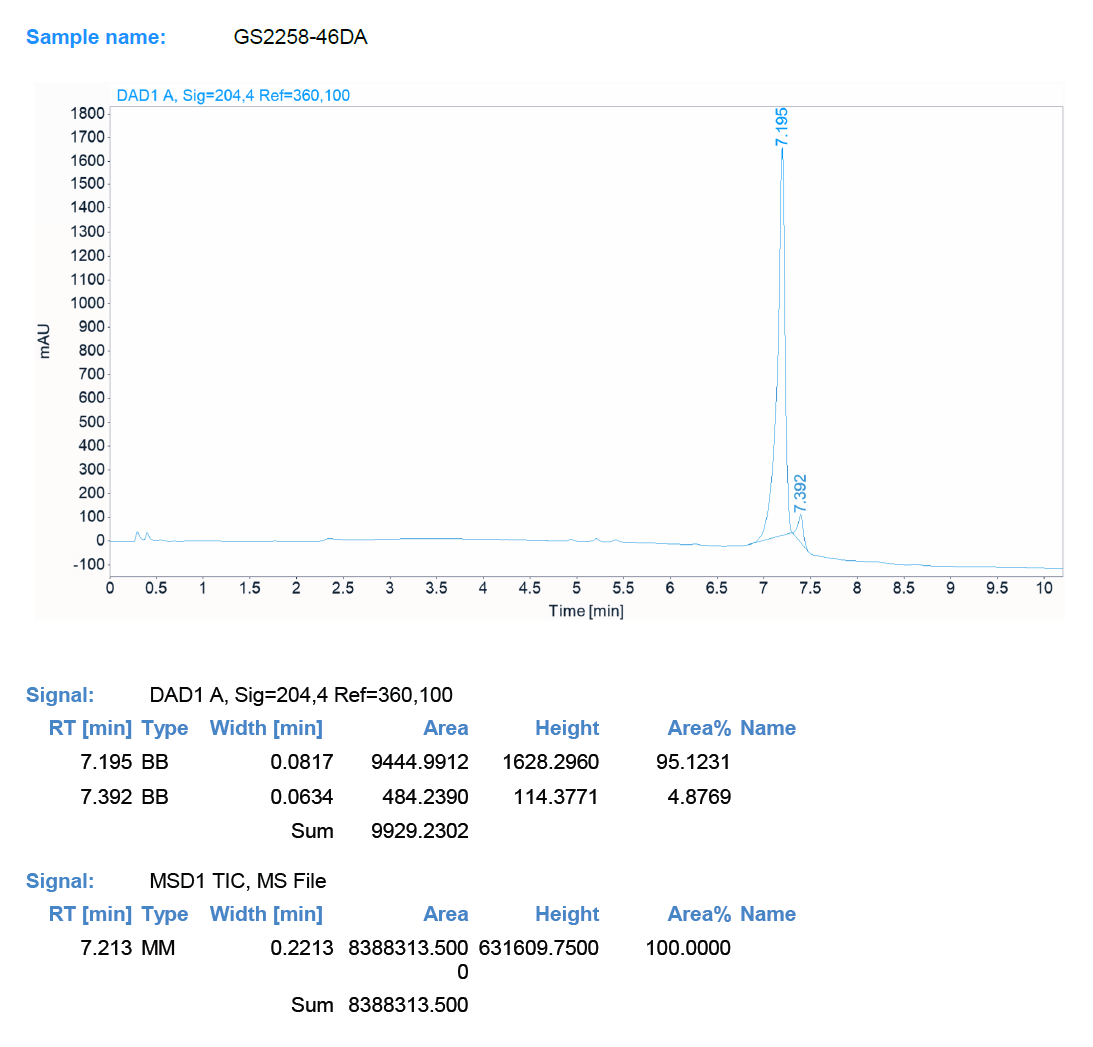


Figure 32: HPLC trace of A1C18_D9_D12


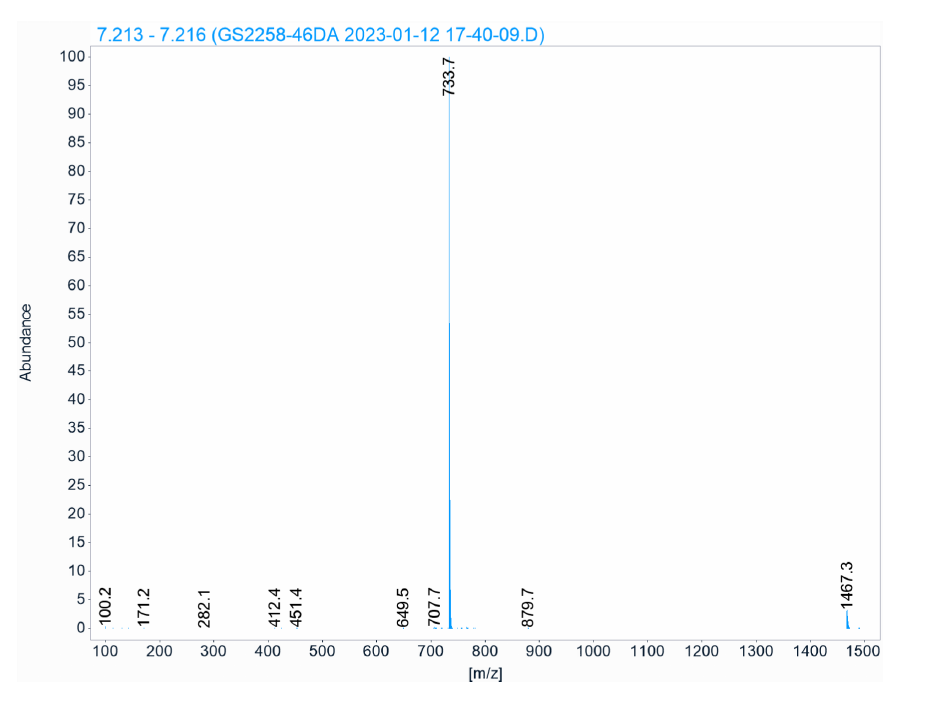


Figure 33: MS spectrum of A1C18_D9_D12


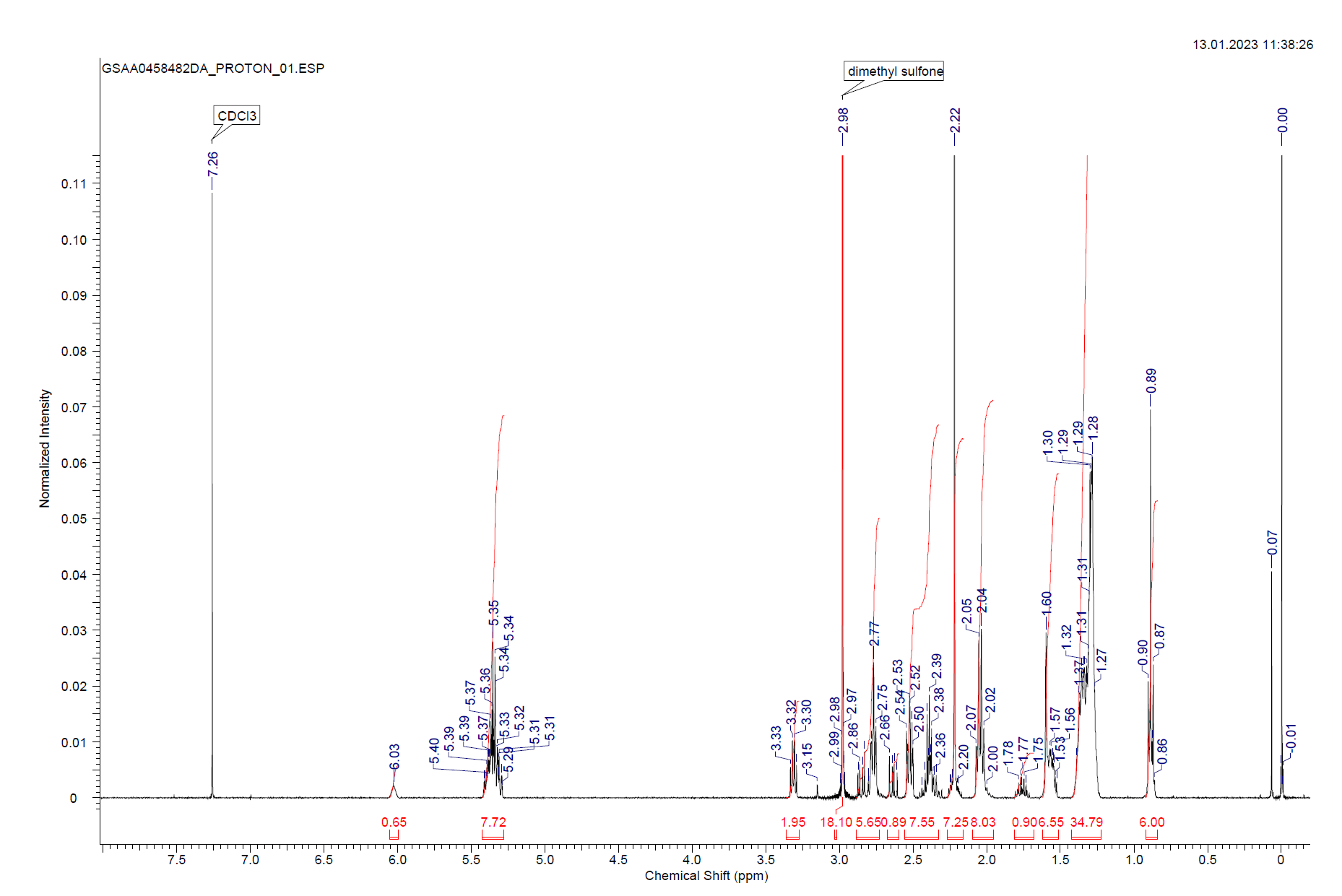


Figure 34: 1H-NMR of compound A1C18_D9_D12


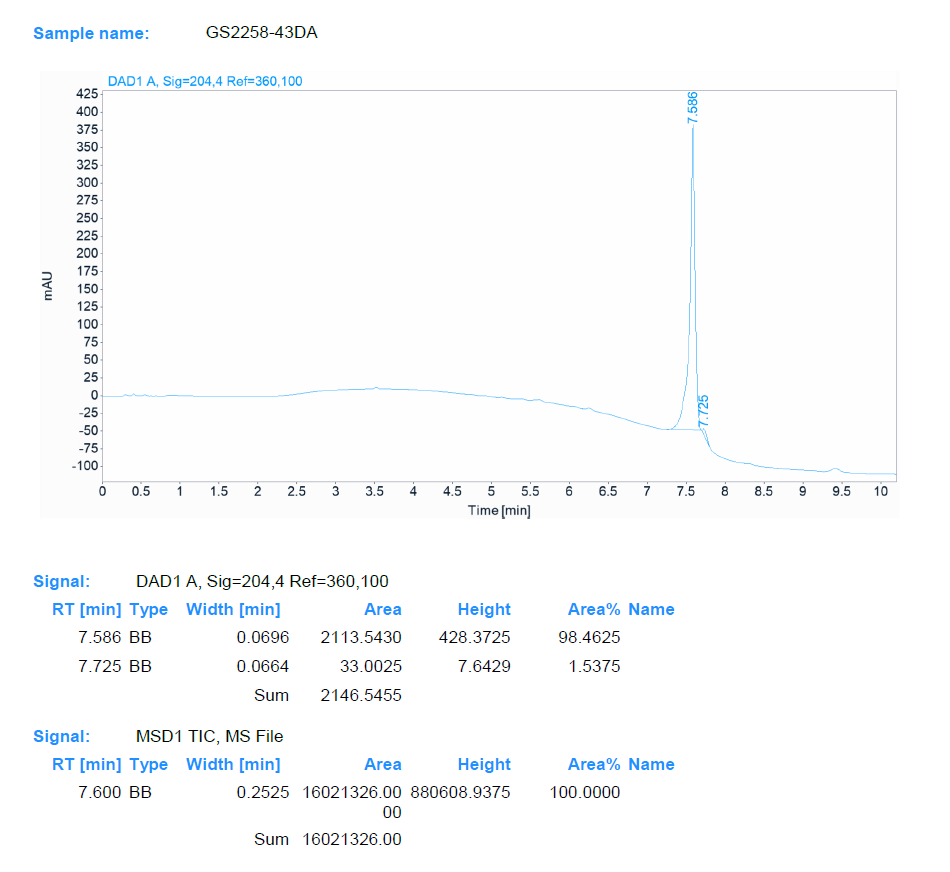


Figure 35: HPLC trace of A1C18_D9


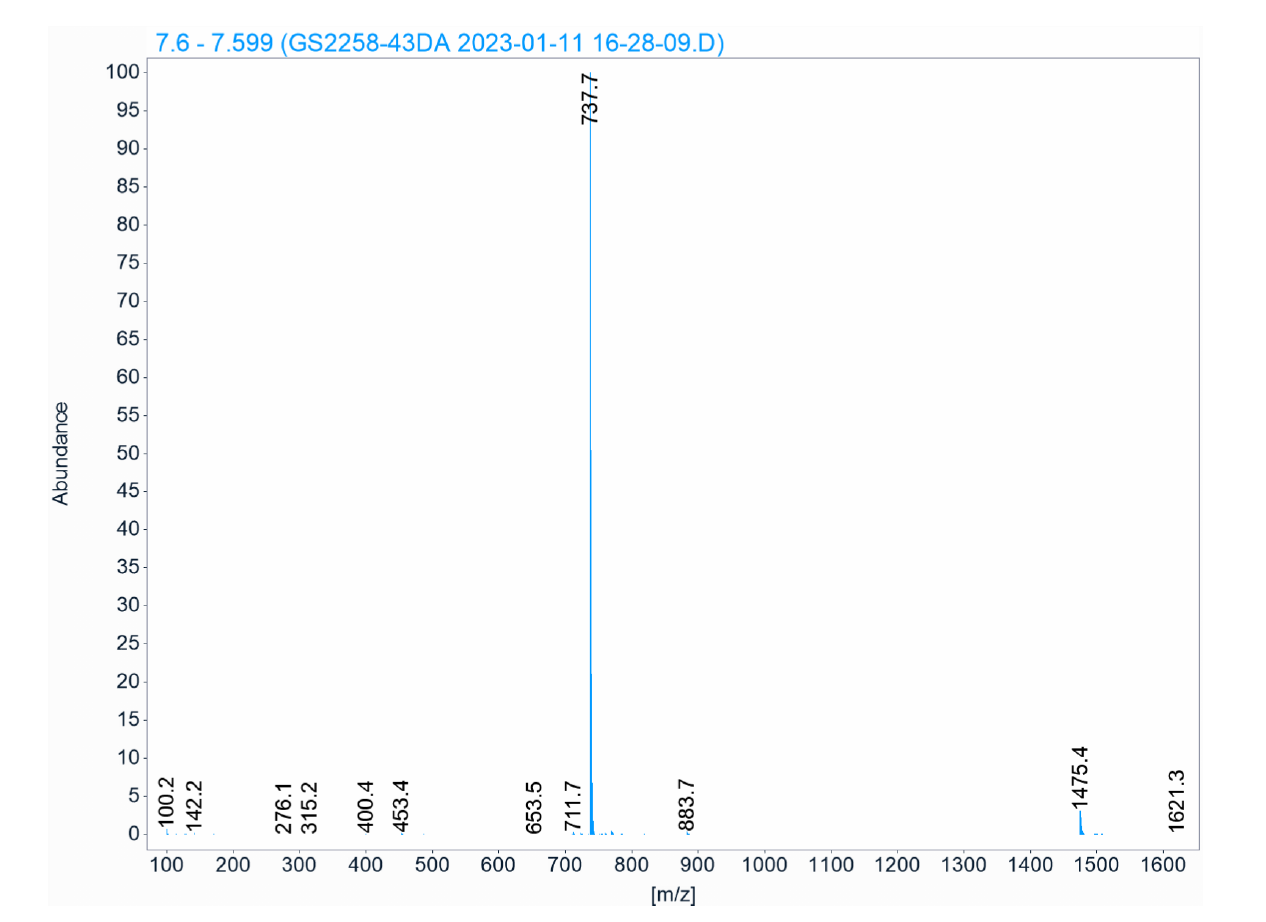


Figure 36: ESI-MS of A1C18_D9


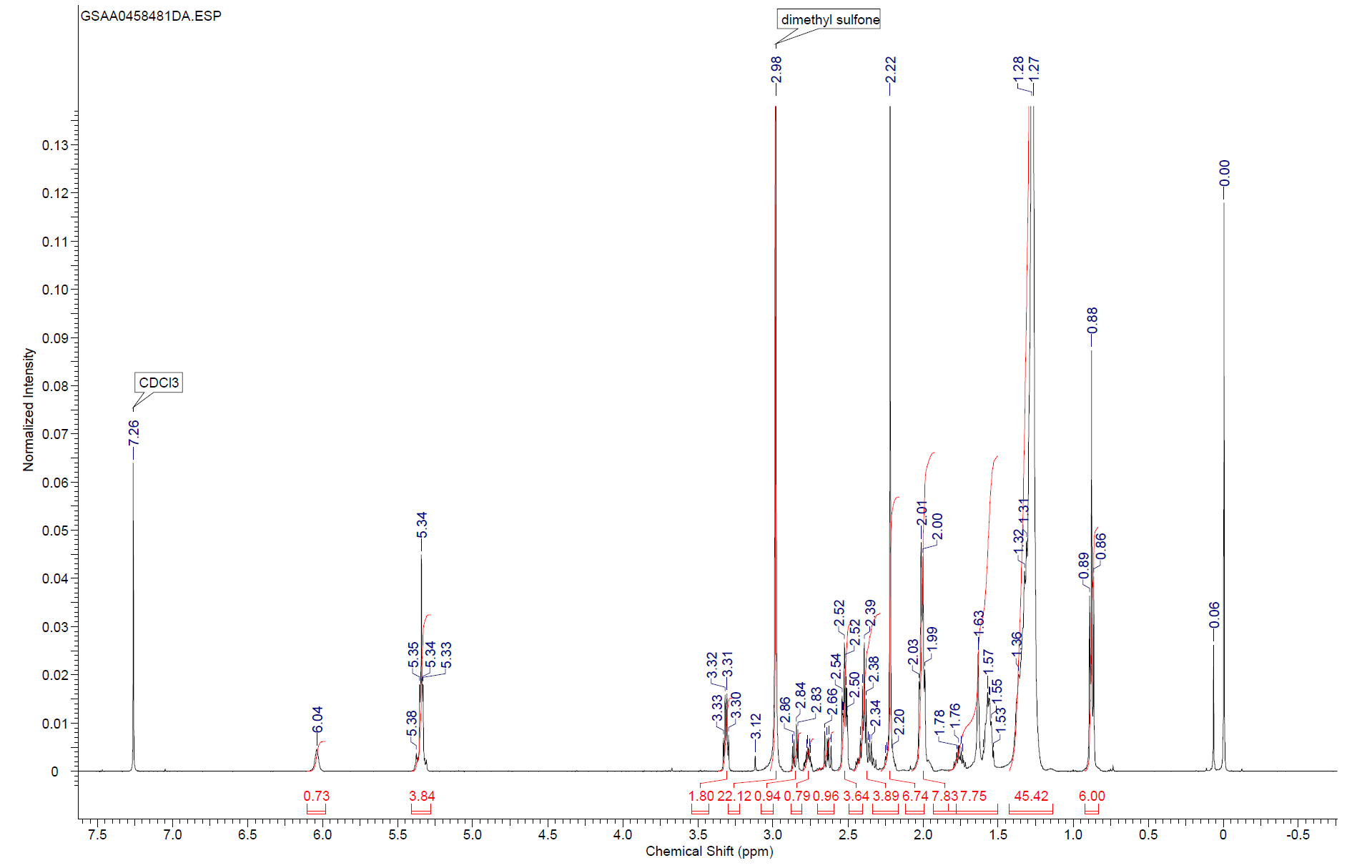


Figure 37: 1H-NMR of compound A1C18_D9


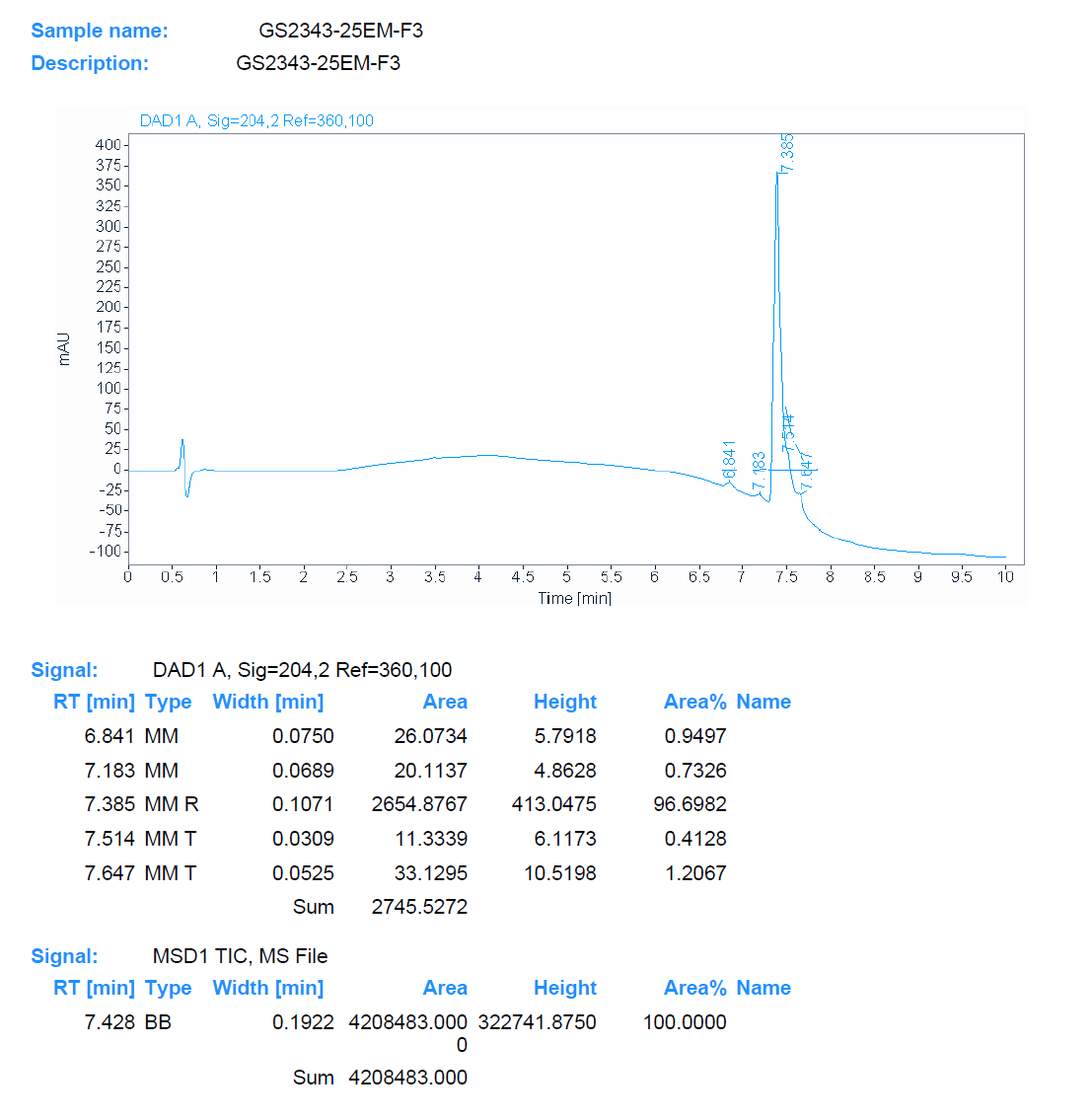


Figure 38: HPLC trace of compound A3C18_D5


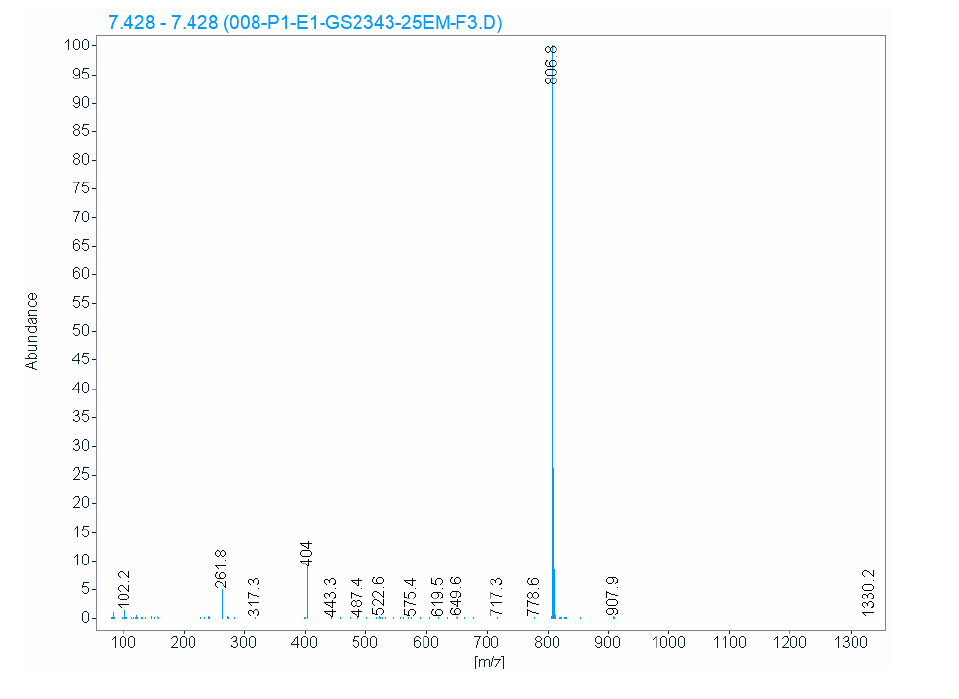


Figure 39: ESI-MS of A3C18_D5


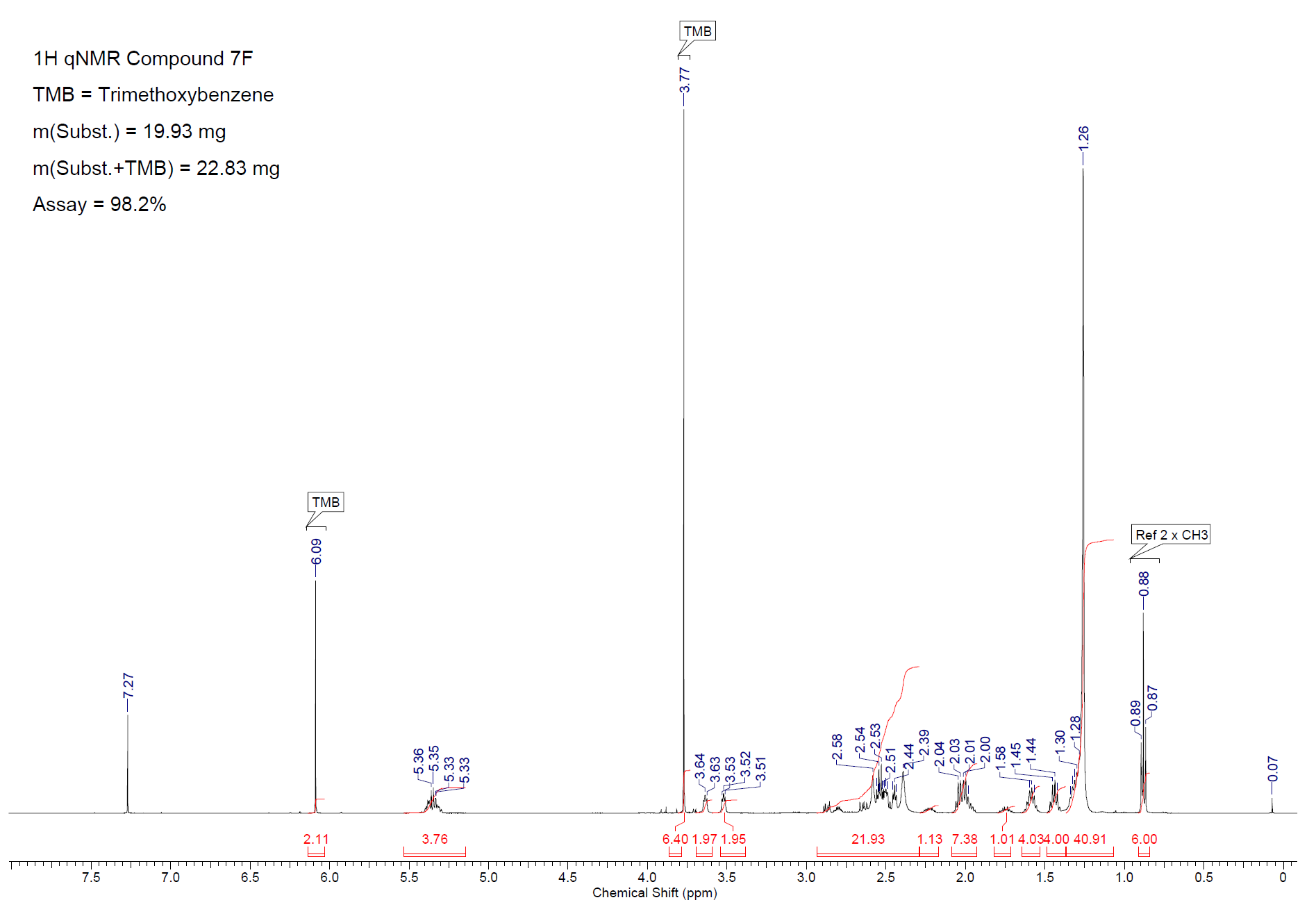


Figure 40: 1H-NMR of compound A3C18_D5


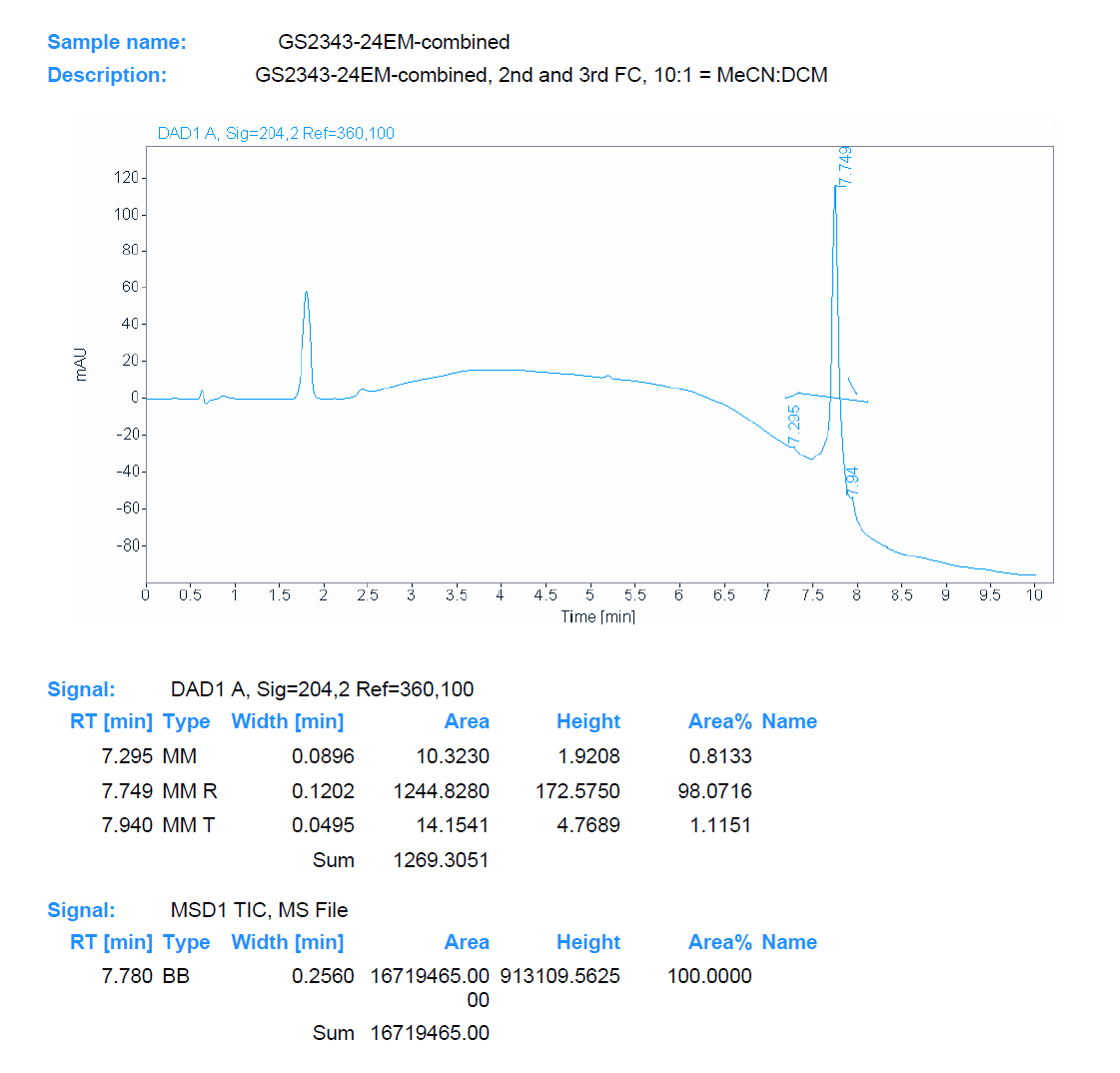


Figure 41: HPLC trace of compound A2C18_D5


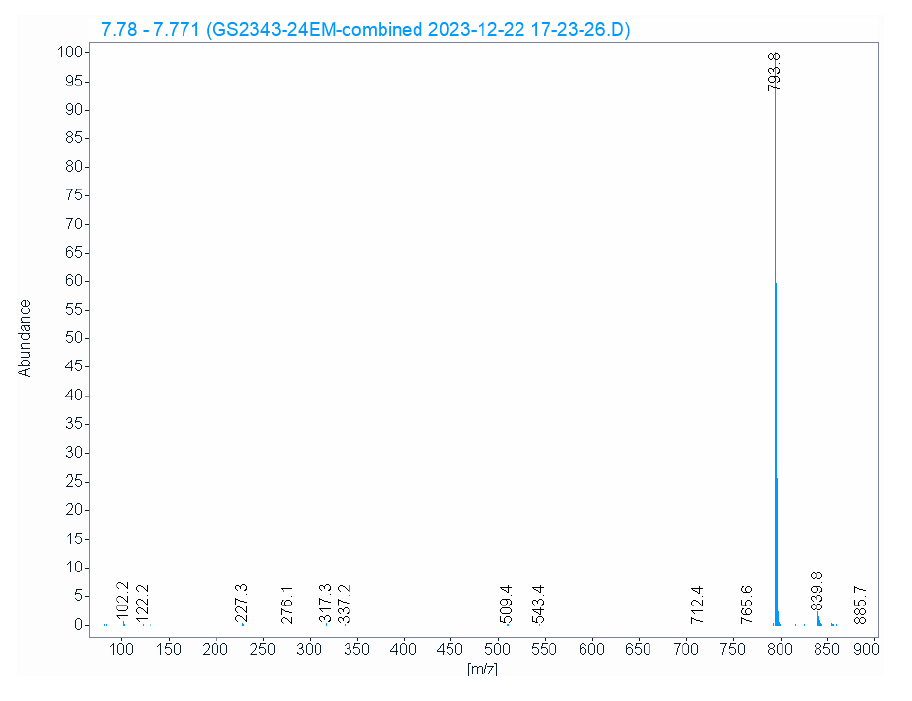


Figure 42: ESI-MS of compound A2C18_D5


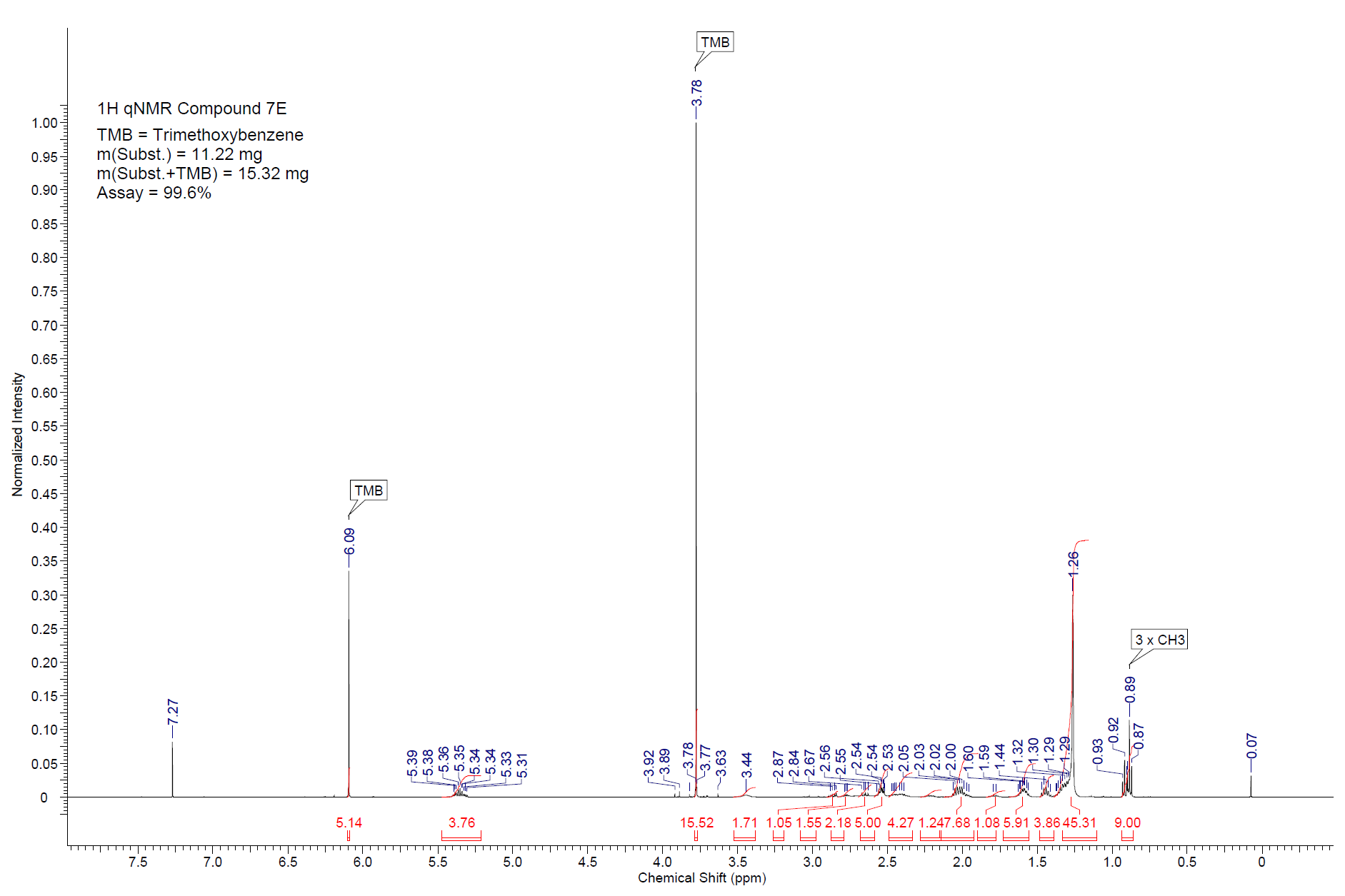


Figure 43: 1H-NMR of compound A2C18_D5


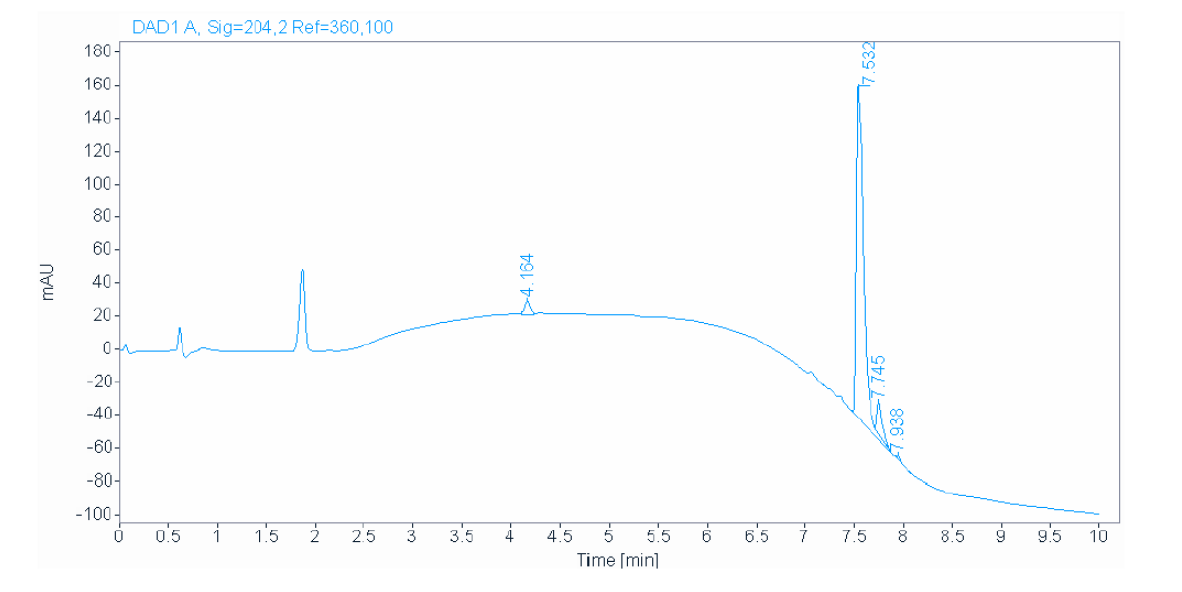


Figure 44: HPLC trace of compound A4C18_D5


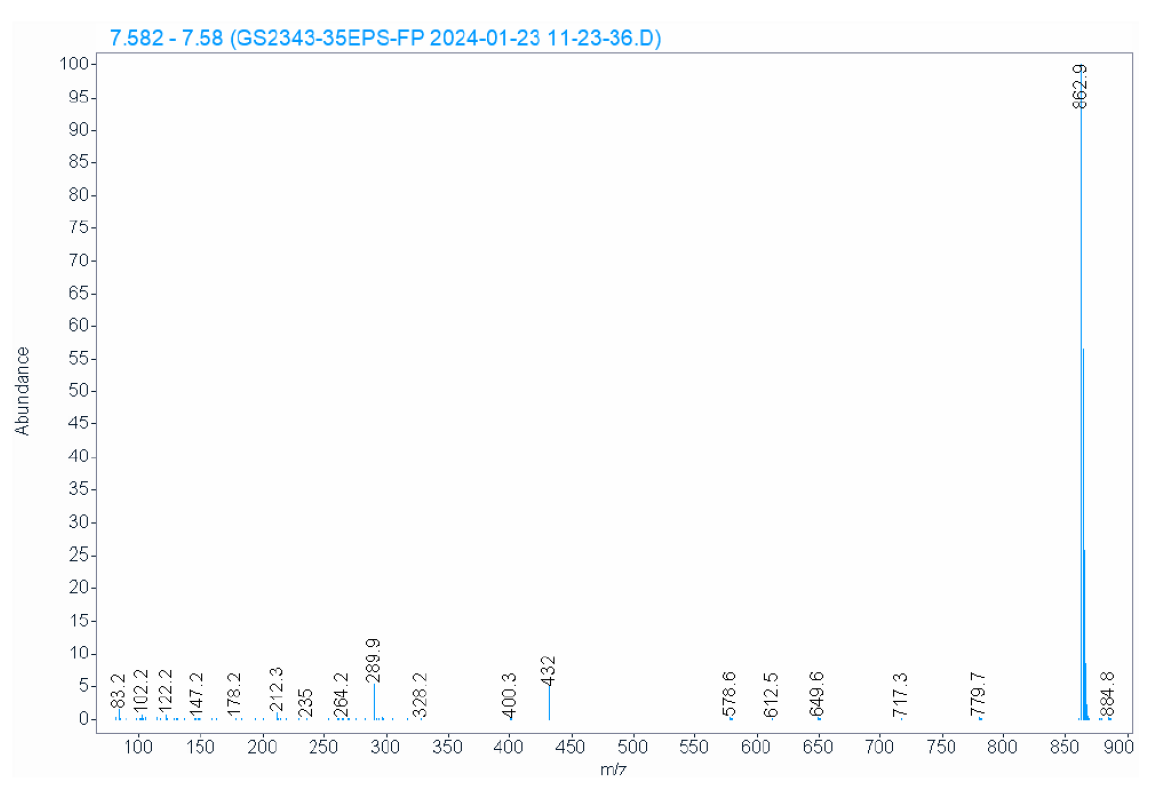


Figure 45: ESI-MS of compound A4C18_D5


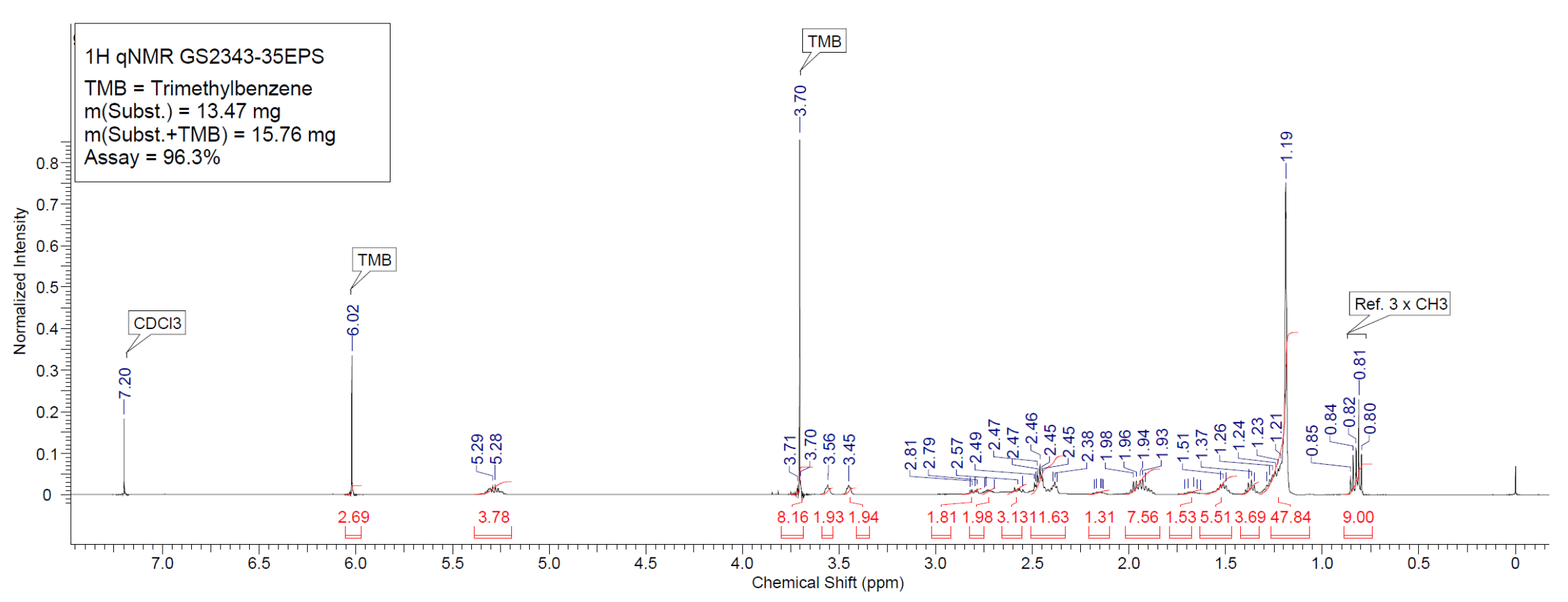


Figure 46: 1H-NMR of compound A4C18_D5


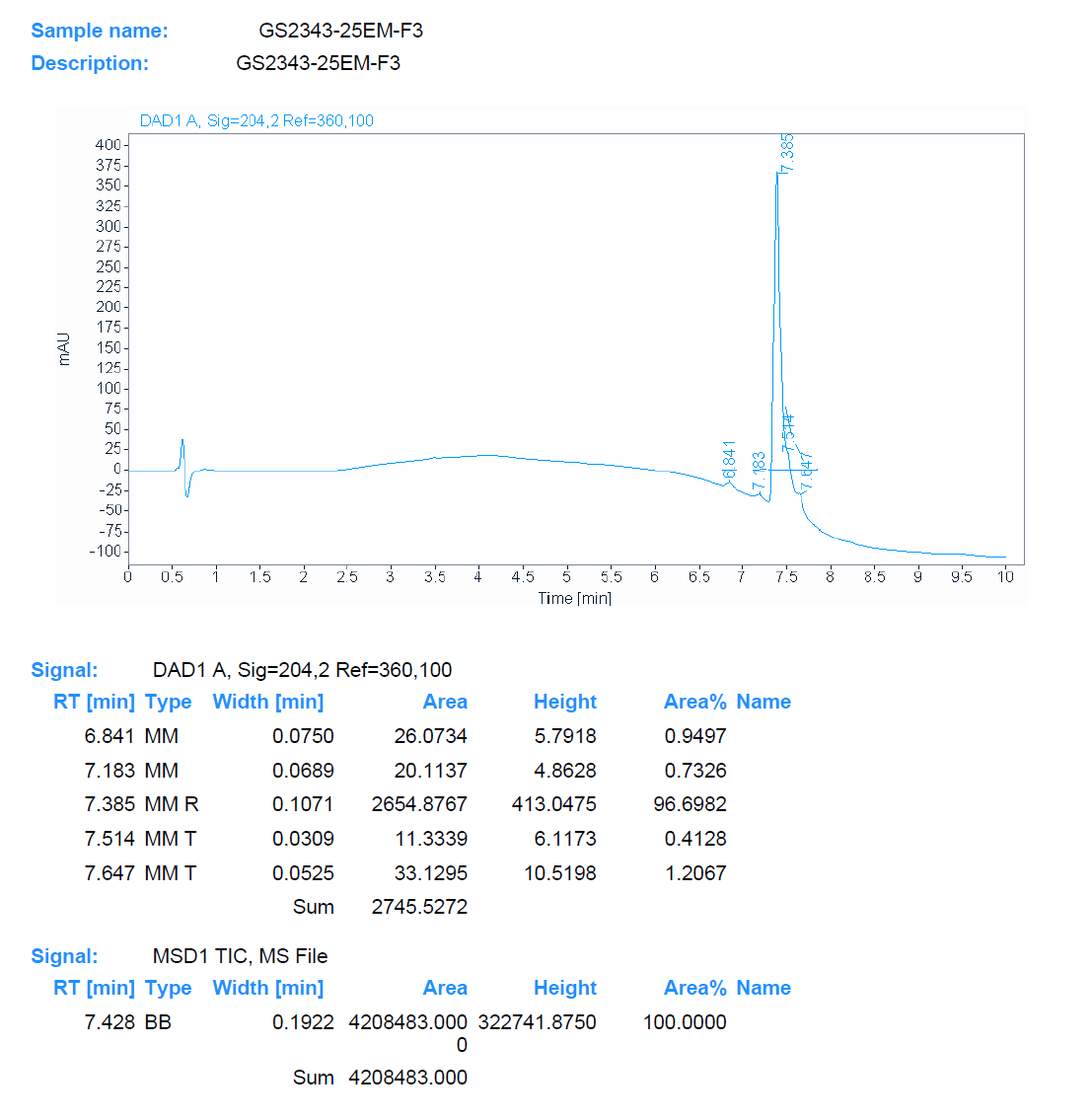


Figure 47: HPLC trace of compound A3C18_D5


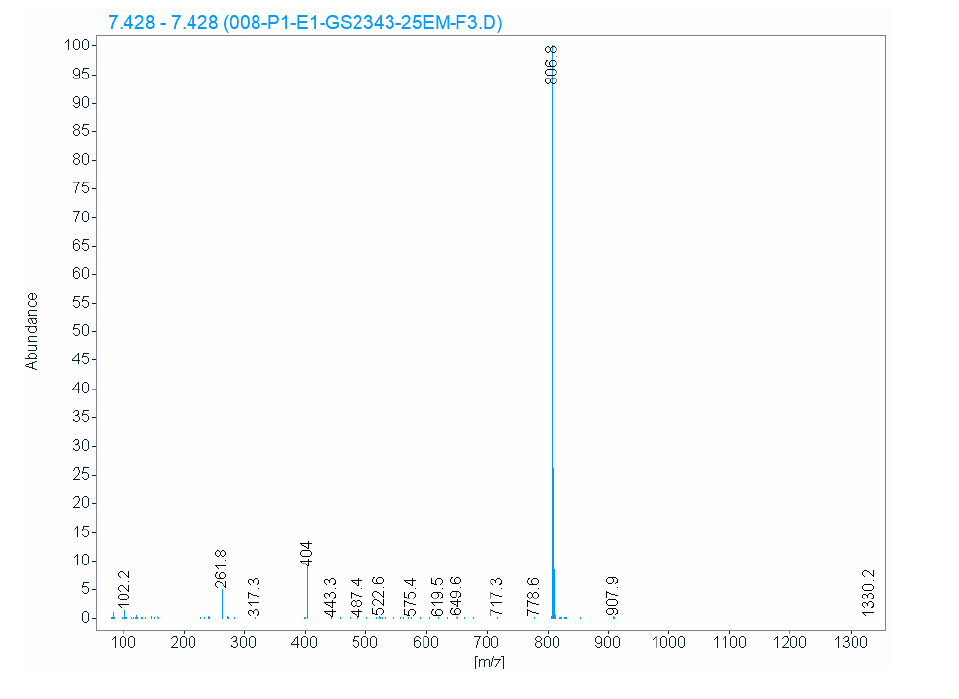


Figure 48: ESI-MS of compound A3C18_D5


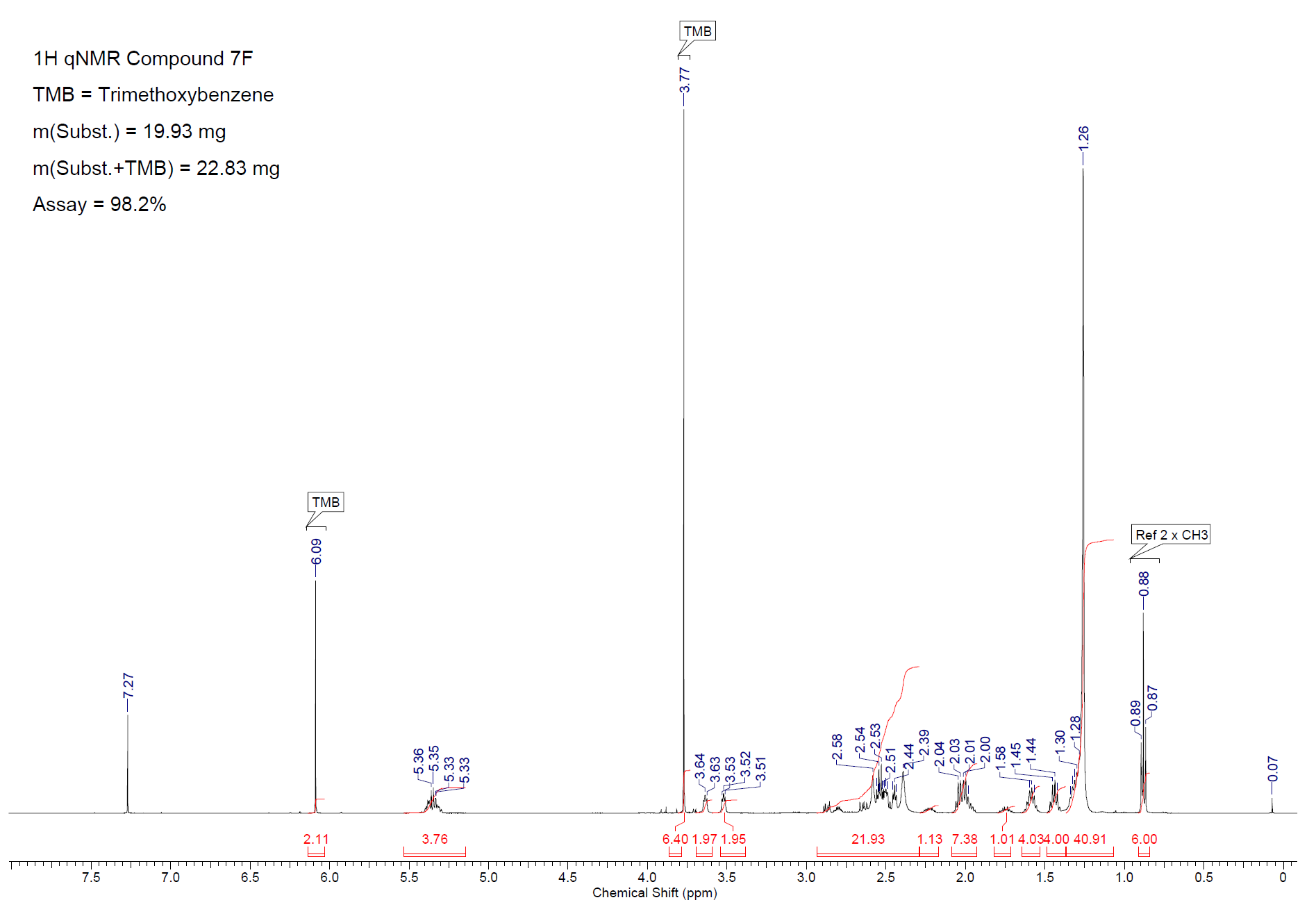


Figure 49: 1H-NMR of compound A3C18_D5


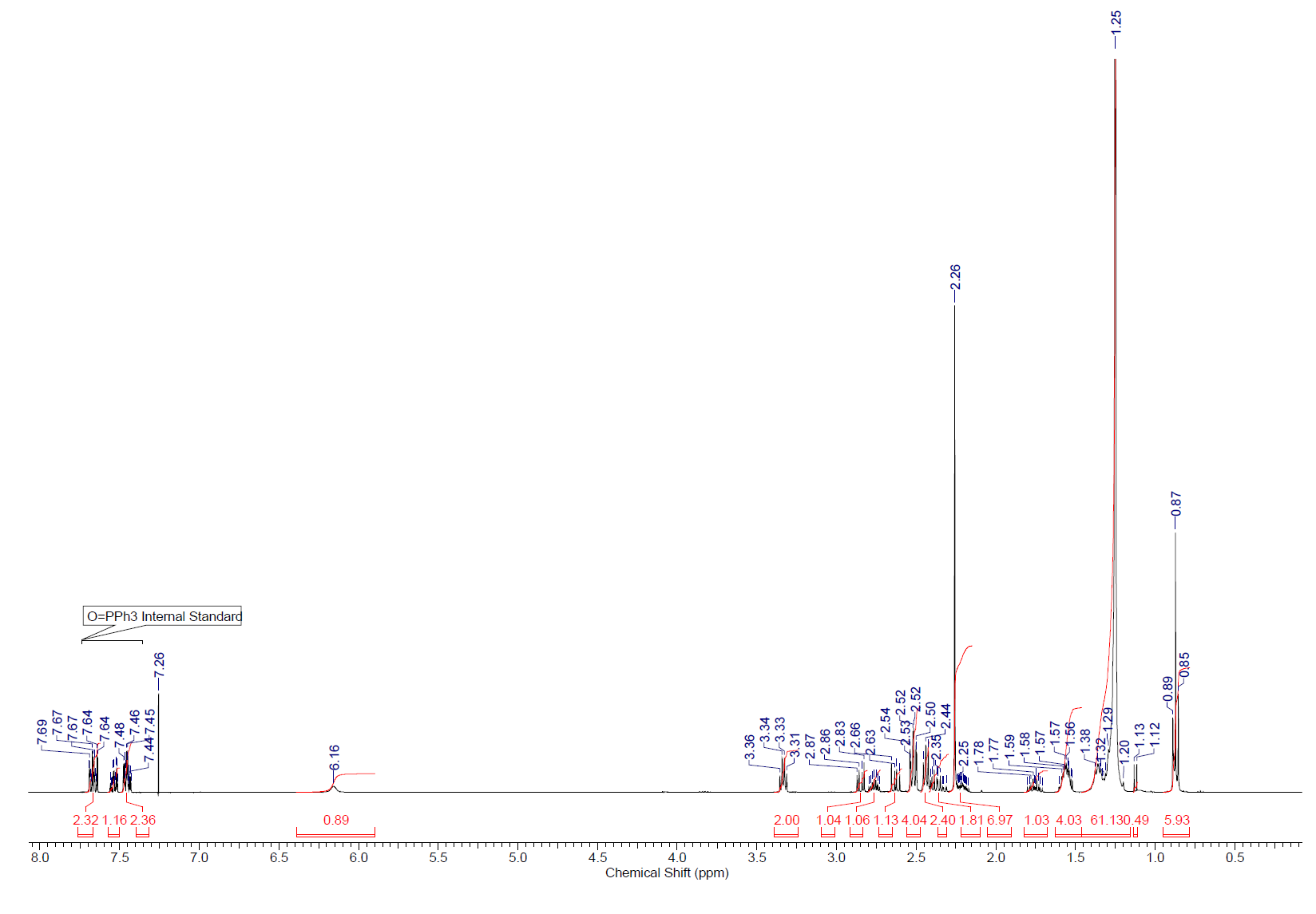


Figure 50: 1H-NMR of compound A1C18


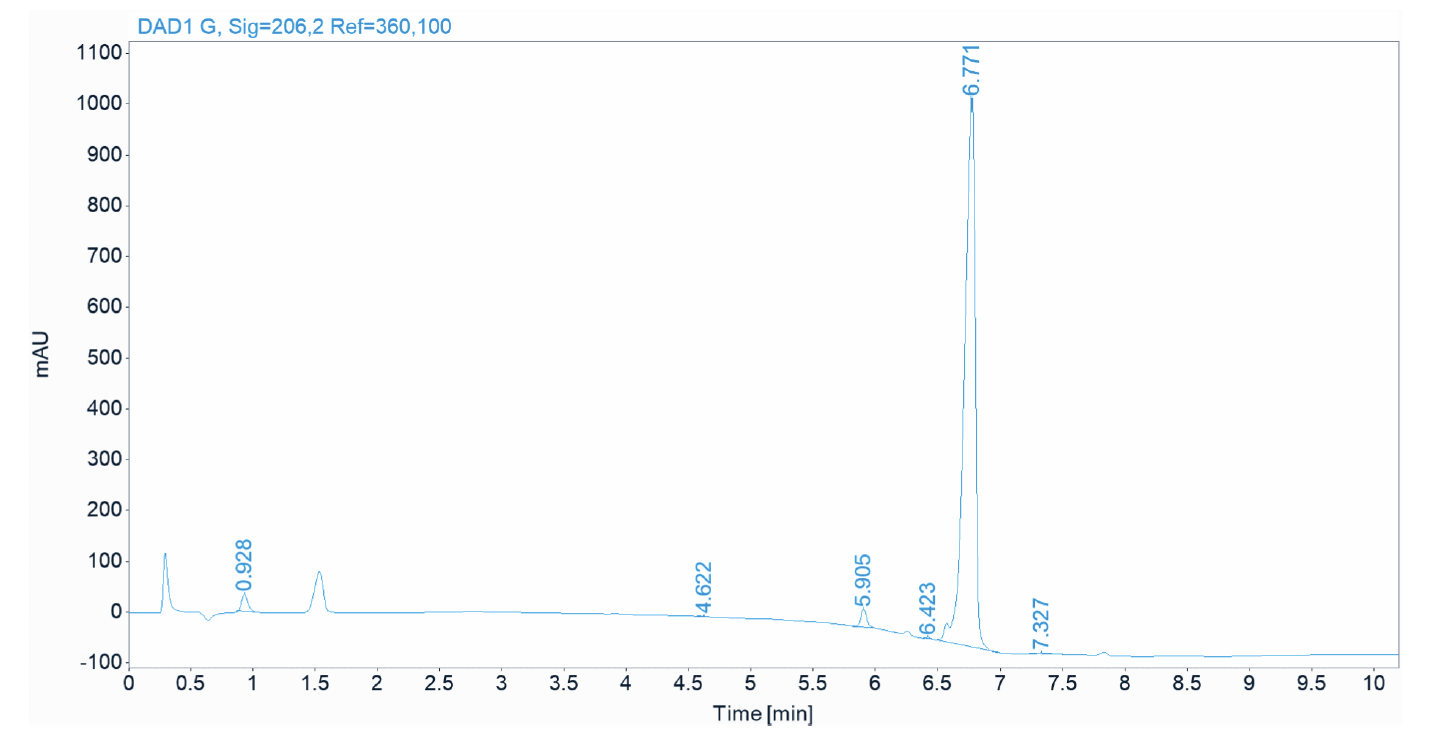


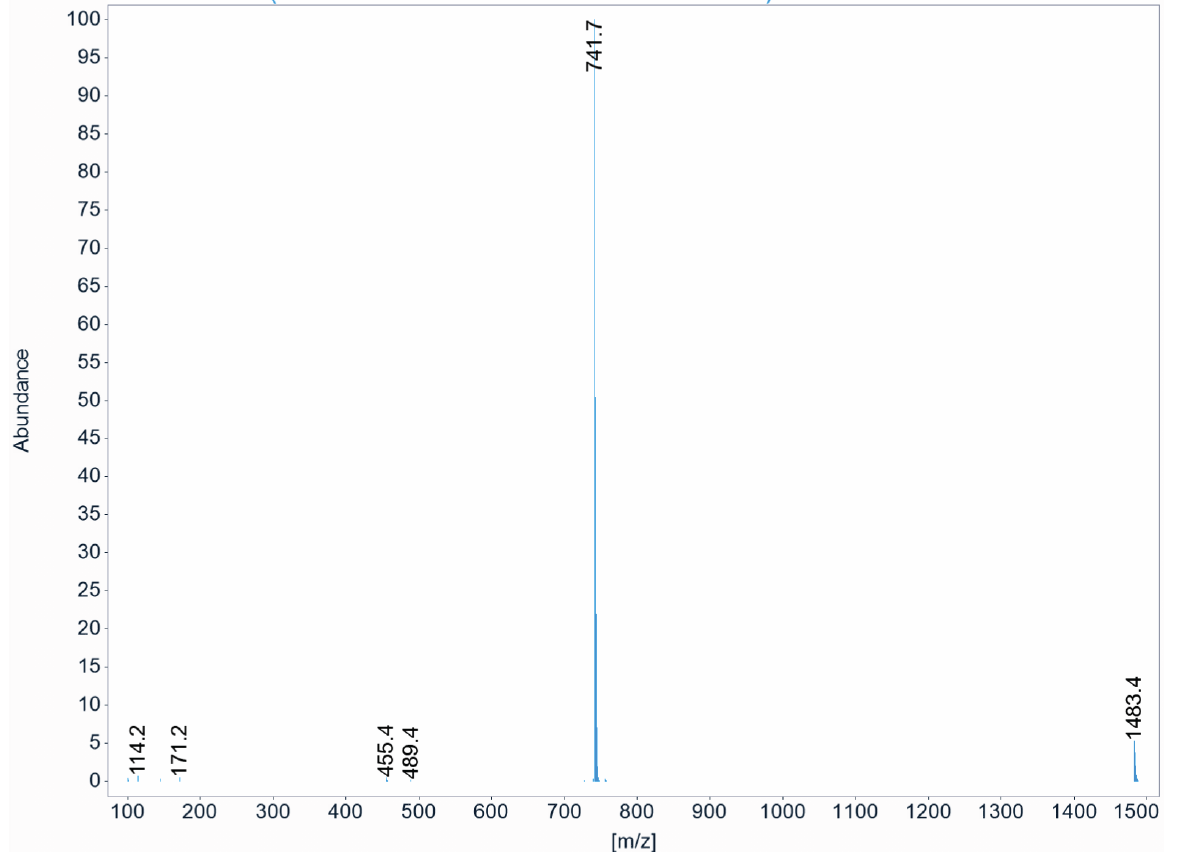


Figure 51: HPLC-MS of compound A1C18


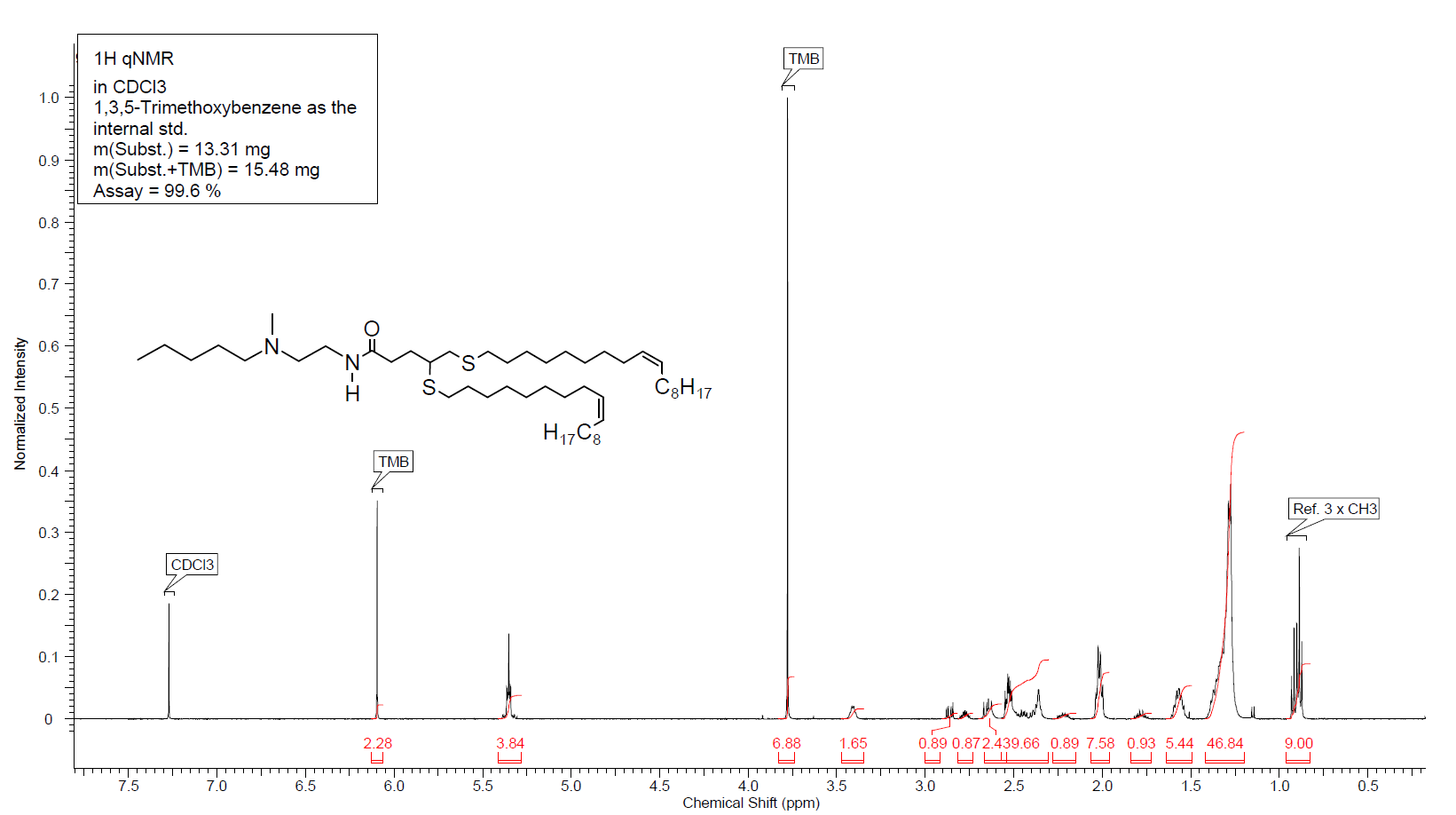


Figure 52: 1H-NMR of compound A2C18_D9


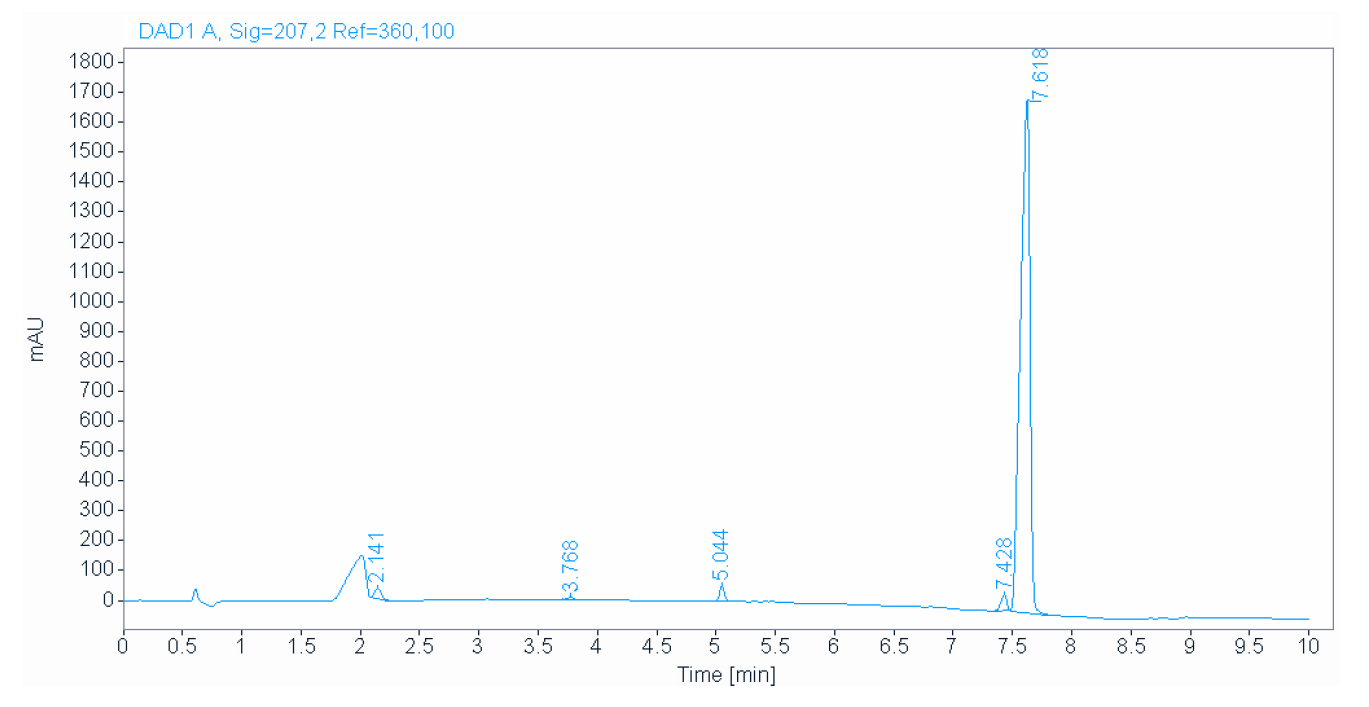


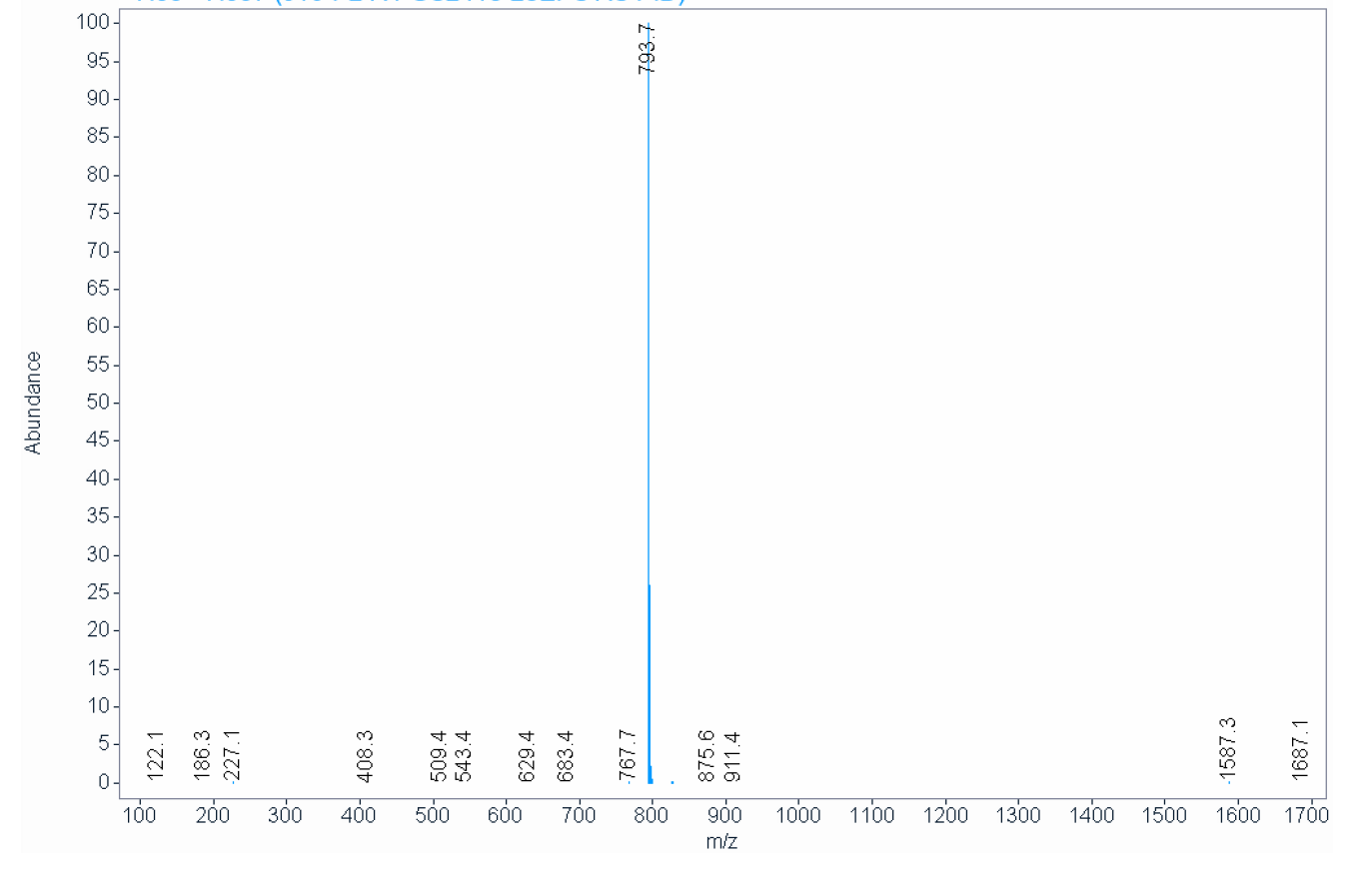


Figure 53: HPLC-MS of compound A2C18_D9


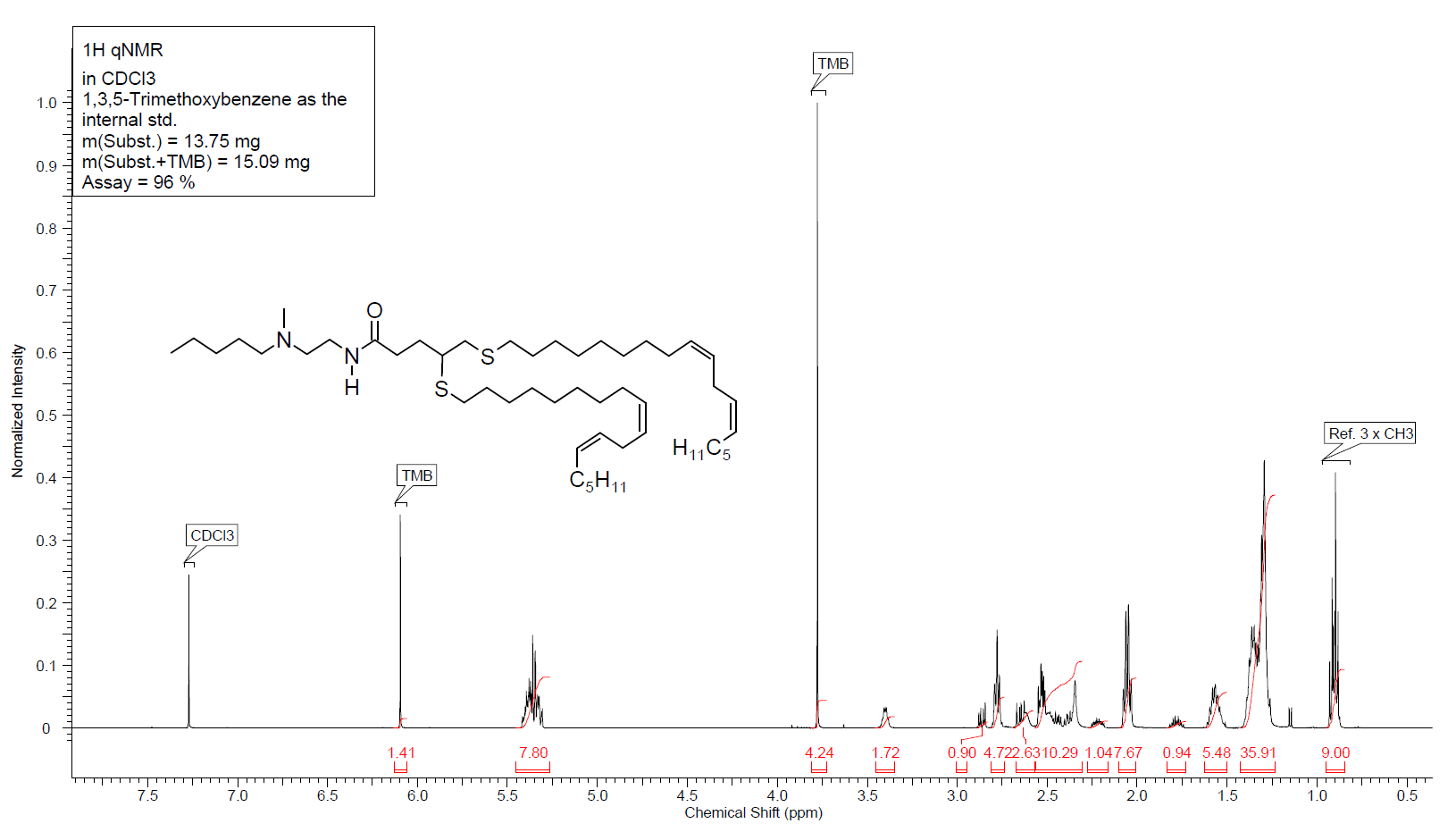


Figure 54: 1H-NMR of compound A2C18_D9_D12


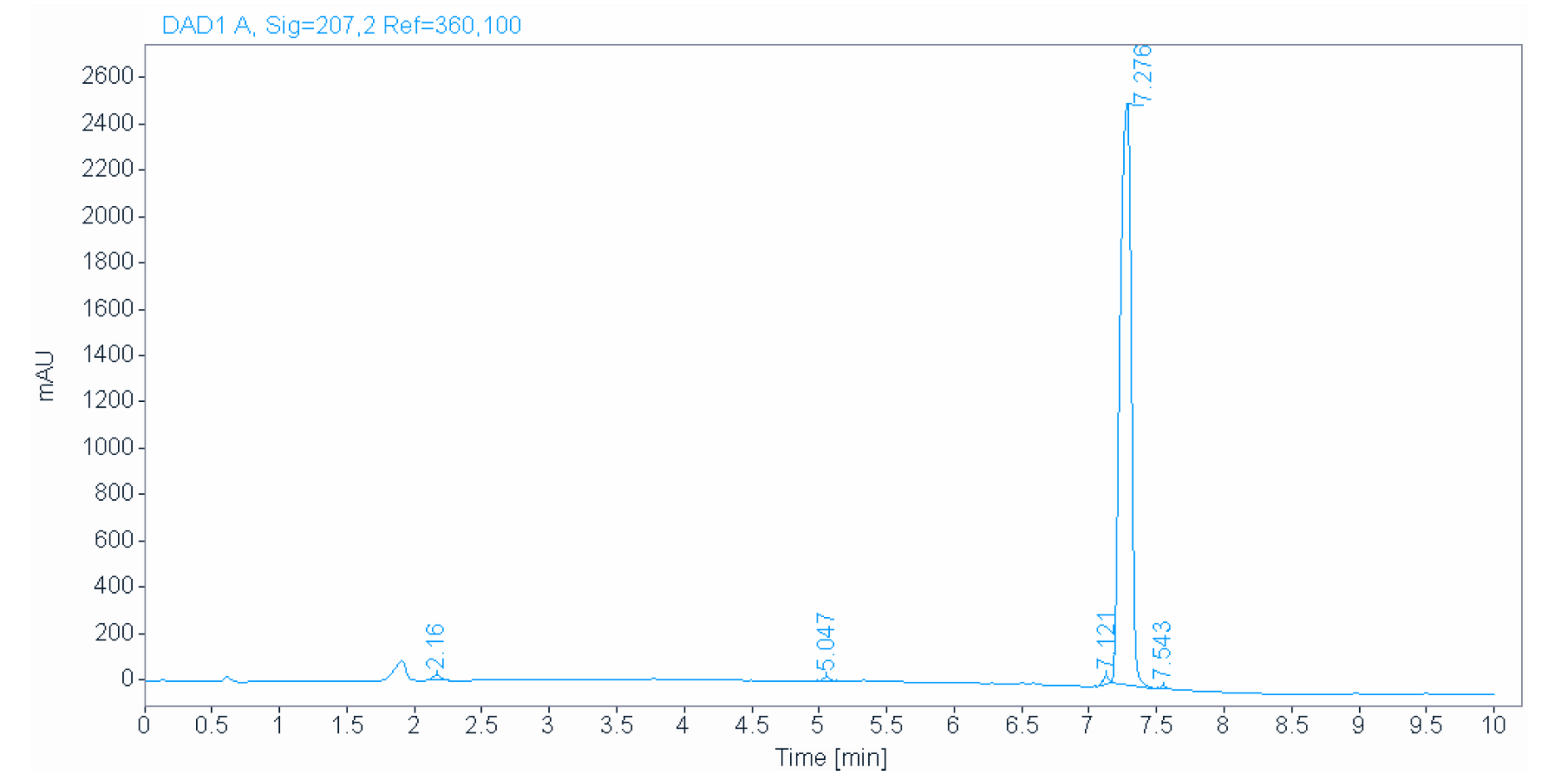


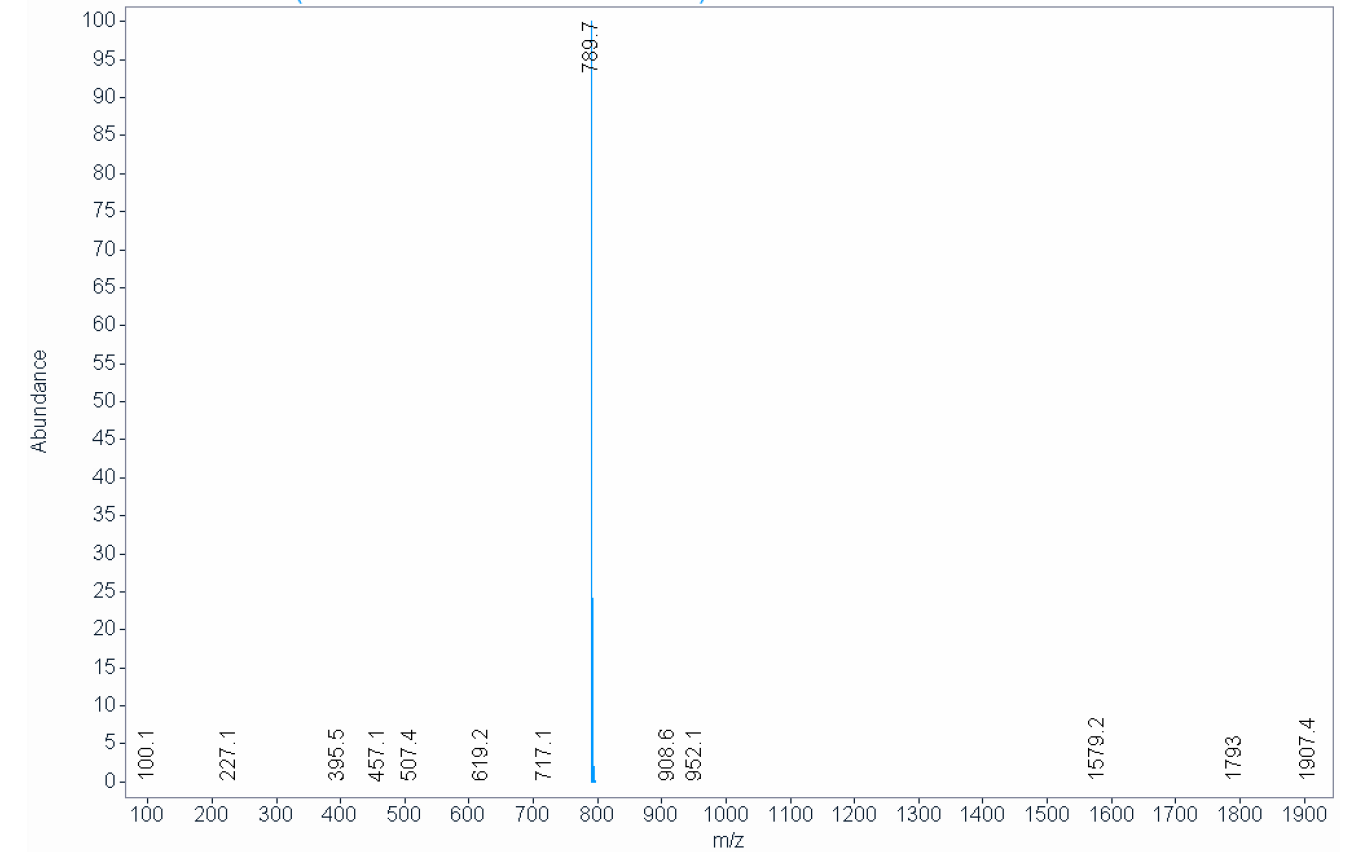


Figure 55: HPLC-MS of compound A2C18_D9_D12


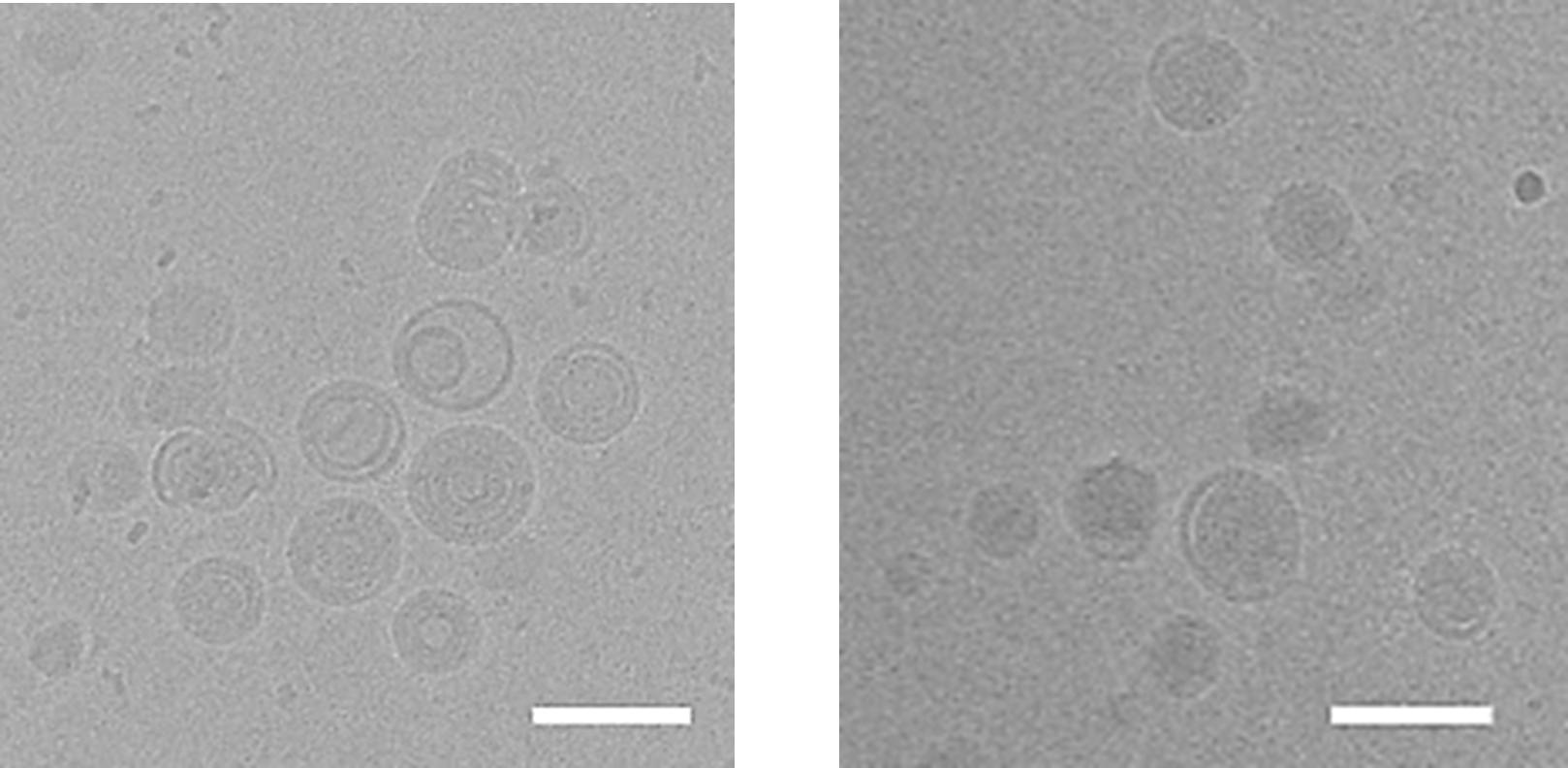


Figure 56: CryoTEM images of LNP containing A1C11 or A2C18_D9 as ionizable lipid. Scale bar shown in white = 100 nm

References

(1) Shepherd, J. N.; Stenzel, J. R. Synthesis of Unsymmetrical Alkynes via the Alkylation of Sodium Acetylides. An Introduction to Synthetic Design for Organic Chemistry Students. *J Chem Educ* 2006, *83* (3), 425. DOI: 10.1021/ed083p425.

(2) Li, L. X.; Zahner, D.; Su, Y.; Gruen, C.; Davidson, G.; Levkin, P. A. A biomimetic lipid library for gene delivery through thiol-yne click chemistry. *Biomaterials* 2012, *33* (32), 8160-8166. DOI: 10.1016/j.biomaterials.2012.07.044.

(3) Schwetlick, K. *Organikum: Organisch-chemisches Grundpraktikum*; Wiley-VCH, 2009.
